# Adherence to inhaled therapies of COPD patients from seven Latin American countries: The LASSYC study

**DOI:** 10.1371/journal.pone.0186777

**Published:** 2017-11-15

**Authors:** Maria Montes de Oca, Ana Menezes, Fernando C. Wehrmeister, Maria Victorina Lopez Varela, Alejandro Casas, Luis Ugalde, Alejandra Ramirez-Venegas, Laura Mendoza, Ana López, Filip Surmont, Marc Miravitlles

**Affiliations:** 1 Universidad Central de Venezuela, Caracas, Venezuela; 2 Post-Graduate Program in Epidemiology, Federal University of Pelotas, Pelotas, Brazil; 3 Universidad de la República, Montevideo, Uruguay; 4 Fundación Neumológica Colombiana, Bogotá, Colombia; 5 Clínica Americana, San José, Costa Rica; 6 Instituto Nacional de Enfermedades Respiratorias, Ciudad de México, México; 7 Hospital Clínico Universidad de Chile, Santiago de Chile, Chile; 8 Hospital Privado Universitario de Córdoba, Córdoba, Argentina; 9 AstraZeneca, Wilmington, Delaware, United States of America; 10 Department of Pneumology, Hospital Universitari Vall d'Hebron, CIBER de Enfermedades Respiratorias (CIBERES) Barcelona, Spain; Lee Kong Chian School of Medicine, SINGAPORE

## Abstract

**Background:**

This study assessed the adherence profiles to inhaled therapies and the agreement between two patient self-report adherence methods in stable COPD lpatients from seven Latin American countries.

**Methods:**

This observational, cross-sectional, multinational, multicenter study involved 795 COPD patients (post-bronchodilator forced expiratory volume in 1 second/forced vital capacity [FEV_1_/FVC] <0.70). Adherence to inhaled therapy was assessed using the specific Test of Adherence to Inhalers (10-item TAI) and the generic 8-item Morisky Medication Adherence Scale (MMAS-8) questionnaires. The percentage agreement and the kappa index were used to compare findings.

**Results:**

59.6% of patients were male (69.5±8.7 years); post-bronchodilator FEV_1_ percent predicted was 50.0±18.6%. Mean values for 10-item TAI and MMAS-8 questionnaires were 47.4±4.9 and 6.8±1.6, respectively. Based on the TAI questionnaire, 54.1% of patients had good, 26.5% intermediate, and 19.4% poor adherence. Using the MMAS-8 questionnaire, 51% had high, 29.1% medium, and 19.9% low adherence. According to both questionnaires, patients with poor adherence had lower smoking history, schooling but higher COPD Assessment Test score, exacerbations in the past-year and post-bronchodilator FEV_1_. The agreement between 10-item TAI and MMAS-8 questionnaires was moderate (Kappa index: 0.42; agreement: 64.7%).

**Conclusion:**

Suboptimal adherence to medication was frequent in COPD patients from Latin America. Low adherence was associated with worse health status impairment and more exacerbations. There was inadequate agreement between the two questionnaires. Greater effort should be made to improve COPD patients’ adherence to treatment, and assessment of adherence with more specific instruments, such as the TAI questionnaire, would be more convenient in these patients.

**Clinical Trial Registration:**

NCT02789540

## Introduction

Chronic obstructive pulmonary disease (COPD) is considered a preventable and treatable disease [1]. The inhaled therapies in COPD have proven to be effective in reducing symptoms, frequency and severity of exacerbations, and improving health status and exercise performance.

Despite the availability of different therapeutic alternatives for COPD, a considerable proportion of patients remain symptomatic or do not achieve treatment goals, which is, in part, due to the poor adherence to inhaled treatment.

Several studies have reported that poor adherence to inhaled therapies is common in COPD with non-adherence being associated with poor symptom control, higher healthcare utilization and costs and decreased health-related quality of life (HRQoL) [2–10]. Other problems in COPD patients related to poor inhalation technique and the use of multiple inhalers requiring different inhalation techniques may influence adherence behavior [11,12].

The Initiative for Chronic Obstructive Lung Disease (GOLD) document specifically recommends evaluation and follow-up of patients’ inhalation technique [1]. However, it does not specify or recommend any method for assessing adherence to inhaled therapy.

Assessing adherence to treatment is complex and many methods have been proposed in COPD (clinician estimates, patient self-reporting, pharmacy records, and electronic monitoring). Patient’s self-report methods accompanied with inhaler technique assessment are considered the most suitable for measuring adherence to medication in clinical practice, even though patients tend to over-estimate adherence.

The 8-item Morisky Medication Adherence Scale (MMAS-8) is a generic self-reported, medication-taking behavior scale that is considered the most commonly used self-reporting method to determine adherence in chronic diseases [13]; however, it was not designed for inhaled medication. In contrast, Plaza et al have recently developed a self-reporting “Test of Adherence to Inhalers (TAI)” questionnaire for assessing inhaler adherence in patients with COPD or asthma [14]. The authors indicate that this is a reliable and homogeneous questionnaire that can be used to identify non-adherence and to classify from a clinical perspective the barriers related to the use of inhalers in asthma and COPD [14].

Limited information exists regarding adherence to inhaled therapies in COPD patients from Latin America, as well as the degree of agreement between different self-report measures in a large COPD population. We hypothesized that adherence to inhaler medication in COPD patients from Latin America is suboptimal and is associated with worse outcomes. Therefore, the aims of this study were to assess the adherence profiles to inhaled therapies and the level of agreement between two patient self-report adherence questionnaires in stable COPD patients from seven Latin American countries.

## Methods

The **L**atin **A**merican **S**tudy of 24-hour **Sy**mptoms in **C**hronic Obstructive Pulmonary Disease (LASSYC) study was conducted in seven Latin American countries: Argentina, Chile, Colombia, Costa Rica, Guatemala, Mexico, and Uruguay. This was a prospective observational, multicenter, multinational, cross-sectional, non-interventional study in stable COPD patients.

Site staff collected, retrospectively, the information from patients’ medical records to determine eligibility. Patients were identified consecutively and, if they meet eligibility criteria and provide consent were enrolled. There was only one scheduled study visit in which the physician explained the patient the purpose of the research and invited to participate. At study visit, selected patients were asked to provide data on disease-related symptomatology assessed during a 24-hour day, adherence to inhalers, HRQoL, and physical activity. The physician collected the following data at visit (from the medical records or interviewing the patient): social demographics, health insurance system, lifestyle, smoking history, comorbidities, level of dyspnea, disease severity, prescribed COPD treatments, exacerbation history, and healthcare resource utilization during the last 12 months.

The study was performed in line with the Declaration of Helsinki and approved by the review board and local ethics committee (the name of the committees in each country are listed in Table A in S1 File), and all patients provided written informed consent.

Inclusion criteria were: male and female outpatients, aged ≥40 years, diagnosis of COPD at least for 1 year, at least one spirometry value with a COPD diagnosis using the post-bronchodilator forced expiratory volume in 1 second/forced vital capacity (FEV_1_/FVC) <0.70 criteria [1] in the previous 12 months, current or ex-smokers (≥10 pack-years), stable disease (without exacerbation treatment and changes in current treatment in the previous 2 months), and signed informed consent. Exclusion criteria were: diagnosis of sleep apnea or any other chronic respiratory disease, any acute or chronic condition that would limit the patient’s ability to participate in the study.

### Assessment of medication adherence

Self-reported medication adherence was measured by the MMAS-8 scale [13] and the TAI questionnaire [14]. Permission to use the MMAS-8 scale and the TAI questionnaire for this research together with the required license agreement was obtained prior to commencing the study. The MMAS-8 scale comprises 8 questions related to medication use as prescribed by the physician. Items 1–7 are Yes/No questions; Yes, is scored as 0 and No as 1 point, except for Question 5 where Yes is scored as 1 point and No as 0. Item 8 is a ranked answer question similar to a Likert Scale across the range 0–4. The total score ranges from 0 to 8. Patient adherence was classified into 3 categories based on the MMAS-8 scale: high adherence (score: 8), medium adherence (score: 7–6), and low adherence (score: <6) [13].

The TAI consists of two complementary questionnaires: the 10-item TAI designed to identify non-adherent patients and to establish the non-adherence level, whereas the 12-item TAI questionnaire (for health care professional) was designed to guide clinically the non-adherence patterns. In the present study, we only use the 10-item TAI questionnaire. This includes (patient domain) self-administered and scored from 1 to 5 (1 = worst possible score; 5 = best possible score). The total score for the 10-item questionnaire ranges from 10–50. Adherence is rated as good (score: 50), intermediate (score: 46–49), or poor (score: <45) [14].

In the 10-item TAI questionnaire, items 1 to 5 allow to identify the erratic non-adherence behavior (forgetfulness to take medication) and items 6 to 10 identify deliberate non-adherence behavior (patient decision not to take medication). The profile of individuals with erratic and/or deliberate non-adherence behavior was assessed following the questionnaire instructions (www.taitest.com), which classified individuals with an erratic non-adherent pattern (sum of items 1–5: <25 points) or in deliberate non-adherent pattern (sum of items 6–10: <25). The 12-item TAI, also includes items #11 and #12 of the health care professional and scored as 1 or 2 (where 1 was bad and 2 was good), with a range from 2 to 4. The latter items were designed to identify two possible causes of unwitting non-adherent behavior. In the present study, these items were not assessed, therefore unwitting non-adherent behavior could not be determined.

### Assessment of early morning, daytime and night-time symptoms

“Evaluating Respiratory Symptoms in COPD” E-RS^™^ 2016 (formerly EXACT-RS) [15] was used to assess daytime symptoms. The night-time and early morning symptoms were assessed with the Nighttime Symptoms of COPD Instrument (NiSCI) [16–18] and Early Morning Symptoms of COPD Instrument (EMSCI) [18].

Dichotomous variables for defining daytime, early morning and night-time symptoms were built. For daytime symptoms, we used the third tertile of the score; the early morning symptoms were defined according to the severity of dyspnea, classified as moderate or higher, plus other symptoms, classified as moderate or more severe; for night-time symptoms, we considered those who woke up at least once at night due to COPD symptoms.

### Statistical analysis

Descriptive statistics included the absolute and relative frequencies for categorical variables and mean and standard deviation for numerical ones. For the comparison between MMAS-8 scale and TAI questionnaires, we used the kappa index. To assess the association between adherence (both scales), and symptoms (continuous score), Poisson regression models were applied. Adjustments took into account sex, age (complete years), pack-years smoked in life, body mass index, airflow obstruction, dyspnea, exacerbations, BODEx index, modified Medical Research Council (mMRC) scale, COPD comorbidity test (COTE), CAT score, physical activity (min/week) and FEV_1_ percent predicted (according to PLATINO equations) [19]. All analyses were performed using Stata 13.1 (StatCorp LP, 2013. Stata Statistical Software: Release 13. College Station, TX, USA).

## Results

**Fig 1:**
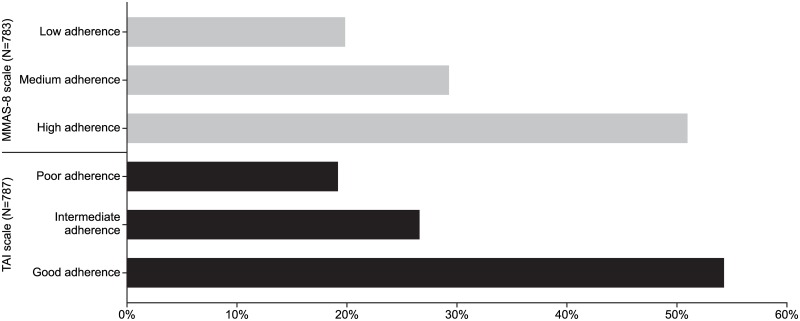
Patient adherence using the MMAS-8 and TAI questionnaires.

795 patients were included, 59.6% were male, mean age of 69.5±8.7 years, with post-bronchodilator FEV_1_ of 50.0±18.6% predicted. Among these patients, 787 (99%) completed the TAI and 783 (98%) the MMAS-8 questionnaire. The proportion of patients in the different categories of adherence according to MMAS-8 and TAI questionnaires is shown in Fig 1. Based on the TAI questionnaire, 54.1% of patients had good adherence, 26.5% intermediate adherence, and 19.4% poor adherence. Using the MMAS-8 questionnaire, 51% had high adherence, 29.1% medium adherence, and 19.9% low adherence.

The sample characteristics of subjects according to MMAS-8 and TAI adherence categories are shown in Tables 1 and 2, respectively. Based on the TAI questionnaire, patients with poor adherence had lower smoking history (pack-years), schooling level and higher CAT score, exacerbations in the past year, and post-bronchodilator FEV_1_ compared with those with good adherence. Similar results were observed using the MMAS-8 questionnaire.

**Table 1:** Patients’ baseline characteristics according to inhaled therapy adherence using the MMAS-8 questionnaire.

Variable	High adherence (N = 399)	Medium adherence (N = 229)	Low adherence (N = 155)	P-value*
**Patient-related factors**				
Sex, male, n (%)	236 (59.2)	133 (58.1)	99 (63.9)	0.491
Age, years, mean (SD)	69.9 (8.8)	69.0 (7.8)	69.4 (9.6)	0.400
BMI, kg/m^2^, mean (SD)	25.9 (5.2)	25.8 (5.1)	25.5 (5.0)	0.797
Smoking history, pack-years, mean (SD)	42.8 (18.9)	41.0 (19.3)	40.7 (19.2)	0.437
Schooling				***0*.*006***
Less than primary	53 (38.7)	48 (35.0)	36 (26.3)	
Finished primary school	95 (48.2)	55 (27.9)	47 (23.9)	
Finished secondary school	143 (58.1)	69 (28.1)	34 (13.8)	
University/college degree	108 (53.2)	57 (28.1)	38 (18.7)	
**Condition-related factors**				
BODEx, mean (SD)	3.0 (1.8)	2.9 (1.9)	2.7 (1.8)	0.171
mMRC scale, mean (SD)	1.8 (1.0)	1.8 (1.1)	1.8 (1.1)	0.936
COTE index, mean (SD)	1.1 (2.2)	1.2 (2.2)	0.9 (1.7)	0.320
CAT, mean (SD)	14.4 (7.8)	15.5 (7.9)	17.0 (8.8)	***0*.*003***
Exacerbation in past year, at least one, n (%)	226 (56.6)	132 (57.6)	106 (68.4)	***0*.*035***
Total physical activity, min/week, mean (SD)	157.1 (258.9)	149.0 (203.8)	133.0 (176.2)	0.538
Number of medicines taken, mean (SD)	2.4 (1.1)	2.4 (1.0)	2.3 (1.1)	0.531
Post-BD FEV_1_, mL, mean (SD)	1179.4 (500.0)	1265.4 (531.6)	1368.6 (553.2)	***<0*.*001***
Post-BD FEV_1_, % predicted, mean (SD)	47.2 (16.6)	50.0 (18.5)	54.2 (17.6)	***<0*.*001***
Post-BD FEV_1_/FVC, %, mean (SD)	47.9 (10.9)	49.6 (11.9)	52.7 (10.9)	***<0*.*001***

* ANOVA for numerical variables and chi-squared test for dichotomous variables. The maximum missing values are for pack-years (n = 120).

BD, bronchodilator; BMI, body mass index; BODEx, body mass index, airflow obstruction, dyspnea, and exacerbations index; CAT, COPD Assessment Test; COTE, COPD specific comorbidity test; FEV_1_, forced expiratory volume in 1 second; FVC, forced vital capacity, mMRC: modified Medical Research Council; SD, standard deviation.

**Table 2:** Patients’ baseline characteristics according to inhaled therapy adherence using the TAI questionnaire.

Variable	Good adherence (N = 427)	Intermediate adherence (N = 209)	Poor adherence (N = 151)	P-value*
**Patient-related factors**				
Sex, male, n (%)	258 (60.4)	125 (59.8)	87 (57.2)	0.833
Age, years, mean (SD)	69.8 (8.4)	69.2 (8.7)	69.1 (9.3)	0.574
BMI, kg/m^2^, mean (SD)	25.8 (5.1)	26.2 (5.1)	25.3 (5.2)	0.288
Smoking history, pack-years, mean (SD)	43.0 (19.1)	42.6 (19.8)	37.7 (17.1)	***0*.*018***
Schooling				***<0*.*001***
Less than primary	53 (38.7)	38 (27.7)	46 (33.6)	
Finished primary school	102 (51.8)	55 (27.9)	40 (20.3)	
Finished secondary school	157 (62.8)	58 (23.2)	35 (14.0)	
University/college degree	115 (56.7)	58 (28.6)	30 (14.8)	
**Condition-related factors**				
BODEx, mean (SD)	2.9 (1.9)	3.0 (1.8)	2.6 (1.8)	0.096
mMRC scale, mean (SD)	1.8 (1.1)	1.9 (1.0)	1.7 (1.0)	0.432
COTE index, mean (SD)	1.2 (2.3)	0.9 (1.9)	1.0 (2.1)	0.208
CAT, mean (SD)	14.2 (7.8)	15.8 (7.8)	17.1 (8.9)	***<0*.*001***
Exacerbation in past year, at least one, n (%)	229 (53.6)	133 (63.6)	102 (67.6)	***0*.*003***
Total physical activity, min/week, mean (SD)	154.8 (243.4)	161.5 (244.9)	127.6 (148.5)	0.344
Number of medicines taken, mean (SD)	2.4 (1.1)	2.4 (1.1)	2.5 (1.1)	0.416
Post-BD FEV_1_, mL, mean (SD)	1226.0 (530.5)	1233.0 (511.2)	1330.8 (540.0)	0.103
Post-BD FEV_1_, % predicted, mean (SD)	48.5 (15.6)	48.4 (16.7)	54.2 (17.9)	***0*.*002***
Post-BD FEV_1_/FVC, %, mean (SD)	48.4 (11.3)	48.2 (11.0)	53.2 (11.3)	***<0*.*001***

* ANOVA for numerical variables and chi-squared for dichotomous ones. The maximum missing values are for packyears (n = 121)

BD, bronchodilator; BMI, body mass index; BODEx, body mass index, airflow obstruction, dyspnea, and exacerbations index; CAT, COPD Assessment Test; COTE, COPD specific comorbidity test; FEV_1_, forced expiratory volume in 1 second; FVC, forced vital capacity, mMRC: modified Medical Research Council; SD, standard deviation.

**Fig 2:**
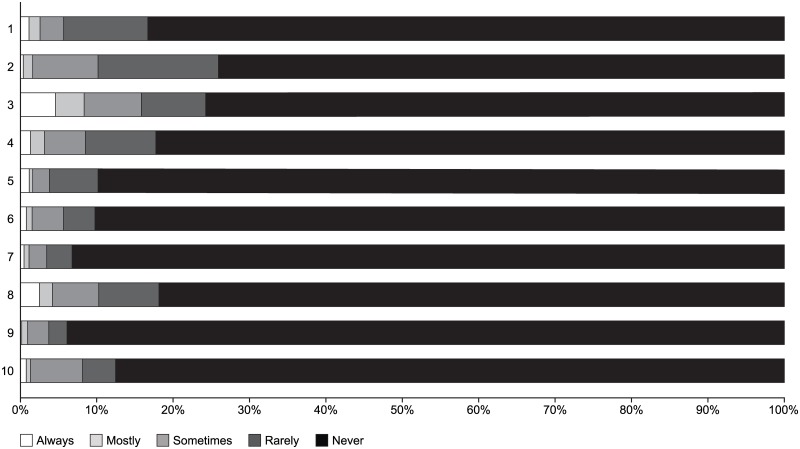
Proportion of patients in each item of the TAI questionnaire according to response type (always, mostly, sometimes, rarely and never).

Mean total and by item values for the TAI questionnaire (47.4±4.9) and MMAS-8 (6.8±1.6) scale are shown in Table 3. The mean TAI score falls in the intermediate adherence category (46–49 points) and the mean MMAS-8 scale value in the medium adherence range (6–7 points). The proportion of patients in each item of the TAI questionnaire according to the response (always, mostly, sometimes, rarely, and never) is shown in Fig 2. The majority of patients (>75%) responded “never” to each question in the TAI questionnaire (Fig 2). Patients’ demographic and characteristic profiles according to non-adherence behavior patterns are shown in Table B in S1 File. A total of 427/787 (54.3%) patients had an adherent pattern, 61/787 (7.8%) had a deliberate non-adherence pattern, 123/787 (15.6%) had an erratic non-adherence pattern, and 176/787 (22.3%) had both erratic and deliberate non-adherence pattern.

**Table 3:** TAI questionnaire and MMAS-8 scale scores (total and by item).

Scale	Item	Mean	SD
**TAI scale**	1	4.74	0.69
	2	4.62	0.73
	3	4.47	1.09
	4	4.69	0.77
	5	4.83	0.59
	6	4.82	0.60
	7	4.88	0.50
	8	4.65	0.87
	9	4.89	0.48
	10	4.77	0.66
	**Total score**	**47.37**	**4.86**
**MMAS-8 scale**	1	0.78	0.41
	2	0.83	0.38
	3	0.90	0.30
	4	0.89	0.32
	5	0.86	0.34
	6	0.92	0.28
	7	0.85	0.36
	8	0.80	0.38
	**Total score**	**6.83**	**1.61**

The agreement between TAI and MMAS-8 questionnaires is shown in Table 4. Based on Cohen’s kappa value (≤0 no agreement, 0.01–0.20 none to slight, 0.21–0.40 fair, 0.41–0.60 moderate, 0.61–0.80 substantial, and 0.81–1.00 almost perfect) [20], the agreement between TAI and MMAS-8 questionnaires was moderate (Kappa index = 0.42; % agreement = 64.7%).

**Table 4:** Comparison of TAI and MMAS-8 scales.

TAI			
MMAS-8	Good adherence	Intermediate adherence	Poor adherence
High adherence	311	63	22
Medium adherence	94	99	34
Low adherence	17	44	91

(Kappa index = 0.42; % agreement = 64.7%)

Crude and adjusted associations between adherence categories (MMAS-8 and TAI scales) with the early morning, daytime and night-time symptoms severity scores and symptoms prevalence are shown in Tables 5 and 6, respectively. Early morning and night-time severity scores were associated with adherence categories (both MMAS-8 and TAI scales) in the crude analysis; however, these did not reach statistical significance in the adjusted analysis (Table 5). Similar trends were observed with symptoms prevalence (Table 6). Tables C and D in S1 File show crude and adjusted association between adherence with CAT score and with exacerbations in the past year, respectively. The CAT score and exacerbations in the past year were associated with adherence categories (both MMAS-8 and TAI scales) in the crude and adjusted analysis.

**Table 5:** Crude and adjusted association between adherence and severity scores of early morning, daytime and night-time symptoms (N = 620, complete information for all variables).

Adherence scale	Mean (SE)	Unadjusted β (95% CI)	Adjusted β (95% CI)
**Night-time severity score**			
*Morisky scale*		*P = 0*.*039*	*P = 0*.*732*
High adherence	2.0 (0.2)	0.0 (ref.)	0.0 (ref.)
Medium adherence	2.7 (0.3)	0.72 (0.05; 1.39)	0.37 (-0.17; 0.91)
Low adherence	2.6 (0.3)	0.64 (-0.09; 1.37)	-0.26 (-0.90; 0.37)
*TAI scale*		*P = 0*.*001*	*P = 0*.*461*
Good adherence	2.0 (0.2)	0.0 (ref.)	0.0 (ref.)
Intermediate adherence	2.4 (0.3)	0.37 (-0.28; 1.03)	-0.15 (-0.69; 0.39)
Poor adherence	3.4 (0.4)	1.39 (0.57; 2.21)	0.33 (-0.35; 1.02)
**Early morning severity score**			
*Morisky scale*		*P = 0*.*032*	*P = 0*.*907*
High adherence	2.9 (0.2)	0.0 (ref.)	0.0 (ref.)
Medium adherence	3.3 (0.3)	0.42 (-0.23; 1.07)	0.04 (-0.49; 0.53)
Low adherence	3.7 (0.3)	0.76 (0.04; 1.48)	-0.05 (-0.65; 0.54)
*TAI scale*		*P = 0*.*006*	*P = 0*.*948*
Good adherence	2.9 (0.2)	0.0 (ref.)	0.0 (ref.)
Intermediate adherence	3.1 (0.3)	0.15 (-0.51; 0.81)	-0.37 (-0.89; 0.14)
Poor adherence	4.1 (0.3)	1.14 (0.39; 1.90)	0.15 (-0.47; 0.78)
**ERS score**			
*Morisky scale*		*P = 0*.*123*	*P = 0*.*344*
High adherence	9.5 (0.4)	0.0 (ref.)	0.0 (ref.)
Medium adherence	10.3 (0.5)	0.80 (-0.52; 2.12)	-0.20 (-1.12; 0.71)
Low adherence	10.5 (0.6)	1.02 (-0.43; 2.47)	-0.48 (-1.48; 0.51)
*TAI scale*		*P = 0*.*021*	*P = 0*.*765*
Good adherence	9.4 (0.4)	0.0 (ref.)	0.0 (ref.)
Intermediate adherence	10.2 (0.6)	0.83 (-0.51; 2.16)	-0.24 (-1.14; 0.67)
Poor adherence	11.1 (0.7)	1.71 (0.20; 3.22)	-0.09 (-1.09; 0.92)

Note: adjusted analyses were performed taking into account sex, age, BMI, smoking history, exacerbations in past year, BODEx index, COTE index, mMRC scale, CAT score, physical activity and FEV_1_% predicted.

**Table 6:** Crude and adjusted association between adherence and prevalence of early morning, day-time and night-time patients’ symptoms (N = 633, complete information on all variables).

Adherence scale	Prevalence (%)	Crude PR (95% CI)	Adjusted PR (95% CI)
**Night time patient**			
*Morisky scale*		*P = 0*.*046*	*P = 0*.*728*
High adherence	15.6	1.0 (ref.)	1.0 (ref.)
Medium adherence	21.3	1.37 (0.94; 1.99)	1.22 (0.87; 1.72)
Low adherence	22.7	1.46 (0.97; 2.20)	1.03 (0.69; 1.52)
*TAI scale*		*P = 0*.*011*	*P = 0*.*486*
Good adherence	16.7	1.0 (ref.)	1.0 (ref.)
Intermediate adherence	16.0	0.95 (0.62; 1.46)	0.82 (0.55; 1.22)
Poor adherence	28.7	1.72 (1.20; 2.46)	1.18 (0.85; 1.64)
**Early morning patient**			
*Morisky scale*		*P = 0*.*078*	*P = 0*.*465*
High adherence	16.5	1.0 (ref.)	1.0 (ref.)
Medium adherence	22;3	1.35 (0.94; 1.95)	1.20 (0.88; 1.64)
Low adherence	22.7	1.37 (0.92; 2.06)	1.11 (0.76; 1.61)
*TAI scale*		*P = 0*.*172*	*P = 0*.*718*
Good adherence	18.2	1.0 (ref.)	1.0 (ref.)
Intermediate adherence	19.6	1.08 (0.74; 1.59)	0.88 (0.62; 1.24)
Poor adherence	24.0	1.32 (0.90; 1.93)	0.95 (0.67; 1.38)
**Day time patient**			
*Morisky scale*		*P = 0*.*200*	*P = 0*.*469*
High adherence	24.5	1.0 (ref.)	1.0 (ref.)
Medium adherence	27.8	1.13 (0.83; 1.54)	0.95 (0.74; 1.23)
Low adherence	30.3	1.25 (0.88; 1.73)	0.90 (0.67; 1.21)
*TAI scale*		*P = 0*.*072*	*P = 0*.*688*
Good adherence	24.1	1.0 (ref.)	1.0 (ref.)
Intermediate adherence	28.9	1.20 (0.88; 1.64)	1.01 (0.77; 1.31)
Poor adherence	31.9	1.33 (0.96; 1.84)	0.94 (0.71; 1.24)

Note: adjusted analyses were performed taking into account sex, age, BMI, smoking history, exacerbations in past year, BODEx index, COTE index, mMRC scale, CAT score, physical activity and FEV_1_% predicted.

## Discussion

The main findings on adherence to inhaled therapies in COPD patients were: first, around 50% of patients had good adherence to inhaled therapies according to TAI and MMAS-8 questionnaires; second, low adherence was associated with lower smoking history, schooling level, worse health status, more exacerbations, and better post-bronchodilator FEV_1_; and third, the agreement between the TAI and MMAS-8 questionnaires was moderate.

Although inhaler therapy is the cornerstone of COPD management, adherence to COPD medication is generally considered to be low, even in very severe disease. The average adherence rate in COPD clinical trials is estimated to be 70–90%; however, in clinical practice, this rate ranges between 20–60%. Few studies have evaluated adherence to inhaled therapies using self-reported methods in COPD. George et al using the medication adherence report scale found self-reported good adherence was present in 37% of the patients [21]. Ágh et al reported that 58.2% had optimal adherence using the MMAS-8 scale [22]. Plaza et al found that adherence to inhaled therapy was higher in COPD patients versus those with asthma (49% vs 28%), with COPD patients having a higher proportion of unwitting non-adherence, and less erratic and deliberate non-adherence than asthma patients [14]. On the other hand, previous studies have shown that the effectiveness of inhaled treatment can be adversely affected by incorrect inhaler technique and prescribing multiple devices requiring different inhalation techniques may lead to poorer outcomes in COPD patients [12]. Our findings are consistent with previous studies as only 54.1% of the patients had good adherence using the TAI questionnaire and 51% had high adherence using the MMAS-8 scale. These results indicate that suboptimal adherence to medication in COPD from Latin America is frequent; therefore, greater efforts must be made to improve adherence in COPD patients from our region. It is important to highlight that we used only the 10-item TAI questionnaire (for identifying non-adherent patients and establish the non-adherence level), so it was not possible to analyze whether patients actually take their inhaler properly or not, as well as to evaluate if the use of multiple devices could influence the adherence level. Therefore, it is not possible to make conclusions on the non-adherence pattern, because it is only available the data on erratic and deliberate non-adherence pattern (unwitting non-adherent behavior data was not collected).

Poor adherence to inhaled therapies is associated with higher morbidity and healthcare utilization, probably as a result of worse and more frequent respiratory symptoms, and more frequent exacerbations [7–9]. Vestbo et al using the TORCH study database reported that of the 4880 patients (79.8%) with good adherence, 11.3% died compared with 26.4% of the patients with poor adherence [10]. The annual hospital admission rates for exacerbations were 0.15 and 0.27, respectively [10]. Others have reported that COPD patients with higher adherence experienced fewer hospitalizations and lower Medicare spending versus lower adherence patients or non-users of maintenance medications [7,8]. A retrospective study involving a large database of insurers in the United States used the proportion of days covered over 12 months to measure adherence and showed that increased adherence to treatment resulted in reductions of health resource use and cost [9].

Most studies that have evaluated the relationship between adherence and outcomes in COPD relied only on pharmaceutical databases, and have had limited clinical practice information. To our knowledge, no information is available regarding the frequency and severity of respiratory symptoms, health status, or exacerbations among real-life COPD patients with high and low adherence to inhaled treatment.

Our results are consistent with those reported in other populations from developed countries showing that patients with low adherence versus those with high adherence had worse health status (CAT score), and more frequent exacerbations in the past year. However, we were unable to find a clear relationship between early morning, daytime and night-time symptoms and treatment adherence. Studies have shown that pharmacologic COPD therapy reduces symptoms, frequency and severity of exacerbations, and improves exercise tolerance and health status; hence, explaining why patients with lower adherence have worse outcomes. Regarding the finding of improved lung function in patients with poor adherence, it is possible that these patients are more likely to have greater erratic and deliberate non-adherence behavior (Table E in S1 File), and regarding the lower smoking history, in patients with poor adherence, we cannot rule out that the smoking finding was an effect of COPD severity. Patients with low adherence smoked less but also had a better FEV_1_ (54.2%) compared with patients with intermediate (50%) and high adherence (47.2%). This would mean that more severe patients are more adherent (probably because they are more symptomatic or experience more relief of symptoms).

The availability of an accurate method to measure inhaled therapy adherence in COPD patients is essential for detecting patients with poor adherence. There are several approaches to detect non-adherence, such as pharmacy refill methods, electronic monitoring (smart-inhaler), and self-report measures, but they are all burdened with important limitations. Recently, several methods to objectively quantify adherence to inhaler therapy (electronic audio recording device) have been assessed [23]. Smart inhalers are emerging as one of the most promising areas to enhance the management and adherence to inhaler therapy. These monitoring devices should be able to monitor adherence, accurately record the time each dose is taken, store data, and provide access to data. Although, the development of these devices would seem interesting for improving inhaler medication adherence, the reality is that their use involves a knowledge and management of technology that is frequently complex for old COPD patients, so this will limit its use to a reduced number of patients.

In the clinical setting, the most convenient approach is using self-report questionnaires because they are easy to use, inexpensive, and not time-consuming. However, compared with data obtained via electronic monitoring, studies have demonstrated that self-reports can be inaccurate because patients generally over-report medication use. To date, there is no “gold standard” self-report questionnaire to assess treatment adherence in COPD patients.

Very few studies have assessed adherence to treatment in COPD using patients’ self-report methods. One study has used two self-report questionnaires [24]; here, there was also a control group (electronic adherence) comprising smartphone user asthma patients using electronic inhaler devices (smart-inhalers). The TAI questionnaire showed a slightly better correlation with adherence determined using Smartinhaler^®^ electronic devices compared with the Morisky-Green test (10-item TAI score: ρ = 0.29; Morisky-Green score: ρ = 0.25) [24]. Regarding the ability to identify adherent and non-adherent patients, the TAI questionnaire showed an intermediate position between the rates observed with the electronic devices and the Morisky-Green test [24].

We assessed the agreement between TAI and MMAS-8 questionnaires (kappa index = 0.42) and found this was moderate (kappa index between 0.41–0.60). Considering that any agreement less than perfect (1.0) is a measure not only of agreement but also of disagreement, any kappa value below 0.60 indicates an inadequate agreement among the measurements. The lack of agreement between the questionnaires may be accounted for by the differences in adherence aspects assessed by each questionnaire and the greater specificity of the TAI questionnaire for the analysis of adherence to inhaled medication versus the generic MMAS-8. The TAI questionnaire not only evaluates the aspects included in the MMAS-8 but also covers a wider range of situations such as forgotten, lack of need of inhaler use, deliberate non-adherent behavior, and concern about side effects and availability [14].

This study has limitations; one is that medication adherence was only assessed using self-reported measurements and this could lead to an over-estimation of medication use. In the present study, we used the 10-items TAI questionnaire so data from the final two items (#11 and #12) of the 12-item questionnaire were not available. These items were designed to identify unwitting non-adherent behavior (failure in understanding medication use, dosage or inhalation technique). Therefore, it was no possible to analyze whether patients take their inhaler properly or not, so that the pattern of non-adherence was not completely evaluated.

In conclusion, our results indicate that suboptimal adherence to medication is frequent in COPD patients from Latin America. Low adherence is associated with worse health status and more exacerbations. There is inadequate agreement between the TAI and MMAS-8 questionnaires, and it would be more convenient to assess adherence to inhaled treatment in COPD with more specific instruments such as the TAI questionnaire.

## Supporting information

S1 File This is the file containing Tables A–E. **Table A**. Patients’ demographics or characteristics according to the non-adherence behavior pattern. **Table B**. Crude and adjusted association between adherence and CAT score (N = 601, complete information for all variables). **Table C**. Crude and adjusted association between adherence and exacerbations in past (N = 602, complete information for all variables). **Table D**. Crude and adjusted association between adherence and exacerbations in past (N = 602, complete information for all variables). **Table E**. Correlation matrix between TAI scale and lung function parameters.
(DOCX)

S2 File LASSYC study protocol.pdf. This is the file containing the study protocol.
(PDF)

S3 File Data set of the individual values of all data presented in this article. (XLSX)
