# Supplementary material for: Adherence to inhaled therapies of COPD patients from seven Latin American countries: The LASSYC study
Source: PLoS One. 2017 Nov 15;12(11):e0186777. doi: 10.1371/journal.pone.0186777 (PMC5687703; doi:10.1371/journal.pone.0186777)
Supplement: S1 File — Table A. Patients’ demographics or characteristics according to the non-adherence behavior pattern. Table B. Crude and adjusted association between adherence and CAT score (N = 601, complete information for all variables). Table C. Crude and adjusted association between adherence and exacerbations in past (N = 602, complete information for all variables). Table D. Crude and adjusted association between adherence and exacerbations in past (N = 602, complete information for all variables). Table E. Correlation matrix between TAI scale and lung function parameters. (DOCX) [file pone.0186777.s001.docx]

**Table A.** Approval committees in the various countries involved in the LASSYC study

| **Country** | **Ethics Committee LASSYC Study** |
| --- | --- |
| Argentina | IRB - Iniciativa y Reflexión Bioética |
| Mexico | LEC: Comité de Etica en Investigacion del Hospital Dr. Angel Leaño  IRB: Comite de Investigacion del Hospital Dr. Angel Leaño |
| Mexico | Comité de Ética en Investigación de México Centre for Clinical Research S.A. de C.V. |
| Mexico | Comité Etica del Hospital General del Estado de Sonora  Institudo Jalisciense de Investigacion Clinica SA |
| Mexico | Winsett Rethman SA de CV |
| Mexico | Comite de Etica en Investigacion de Mexico. Centre for Clinical Research, SA de CV |
| Mexico | Instituto Jaliscience de Investigacion Clinica SA de CV (IJICSA) |
| Mexico | Comité de Etica en Investigacion  Comité de Investigacion del Hospital Angeles de Las Lomas |
| Chile | Comité de Etica del Servicio de Salud Metropolitano Sur Oriente |
| Chile | Comité Etico Científico del Servicio de Salud Metropolitano Oriente (RC)  Comité de ética del Hospital Clínico de la Fuerza Aérea de Chile |
| Colombia | Comité de Etica en la Investigacion CAIMED |
| Colombia | Comité de Etica en Investigación Fundación Neumológica Colombiana (CEI-FNC) |
| Colombia | Comité Corporativo de Etica (CCEI) |
| Costa Rica | Comité ético Científico UCIMED |
| Guatemala | Comité de Ética Independiente Zugueme |

**Table B.** Profile of individuals according to the non-adherence behavior patterns

|  |  | **Adherent pattern (n=427)** | **Intentional or deliberate non-adherence pattern (n=61)** | **Erratic non-adherence pattern (n=123)** | **Both erratic and intentional/deliberate non-adherence pattern (n=176)** | **p value** |
| --- | --- | --- | --- | --- | --- | --- |
| Sex (male) | N (%) | 258 (60.4) | 34 (55.7) | 80 (65.0) | 98 (55.7) | *0,372* |
| Age, years | Mean (SD) | 69.8 (8.4) | 68.6 (9.7) | 69.8 (8.3) | 68.9 (9.2) | *0,528* |
| FEV_1_ (post-BD, % predicted) | Mean (SD) | **48.5 (17.6)** | **43.4 (14.8)** | **52.2 (16.9)** | **52.3 (18.1)** | ***0,001*** |
| FVC (post-BD, % predicted) | Mean (SD) | 72.8 (19.1) | 70.0 (17.5) | 75.0 (19.3) | 74.2 (18.6) | *0,292* |
| FEV_1_/FVC % | Mean (SD) | **48.4 (11.3)** | **45.9 (11.7)** | **50.8 (10.3)** | **51.7 (11.6)** | ***<0.001*** |
| mMRC grade | Mean (SD) | 1.8 (1.1) | 2.0 (0.9) | 1.8 (1.1) | 1.8 (1.0) | *0,649* |
| CAT score | Mean (SD) | **14.2 (7.8)** | **17.0 (8.4)** | **15.0 (7.8)** | **17.0 (8.4)** | ***<0.001*** |
| BODEx index | Mean (SD) | 2.9 (1.9) | 3.4 (1.8) | 2.7 (1.7) | 2.7 (1.8) | *0,088* |
| Day time symptoms score | Mean (SD) | **9.4 (6.7)** | **10.9 (8.2)** | **10.1 (6.9)** | **11.1 (7.1)** | ***0,034*** |
| Early morning symptoms score | Mean (SD) | **2.9 (3.4)** | **3.7 (3.9)** | **2.9 (3.2)** | **4.0 (3.7)** | ***0,005*** |
| Night time symptoms score | Mean (SD) | **1.9 (3.4)** | **2.4 (3.9)** | **2.0 (3.0)** | **3.3 (4.1)** | ***<0.001*** |

Note: for sex, the p-value was obtained using chi-square test. For other variables, ANOVA was used

**Table C.** Crude and adjusted association between adherence and CAT score (N=601, complete information for all variables)

| **Adherence scale** | **Mean (SE)** | **Unadjusted β (95% CI)** | **Adjusted β (95% CI)** |
| --- | --- | --- | --- |
| *Morisky scale* |  | *P=0.002* | *P=0.007* |
| High adherence | 14.4 (0.5) | 0.0 (ref.) | 0.0 (ref.) |
| Medium adherence | 16.0 (0.6) | 1.63 (0.16; 3.11) | 0.87 (-0.12; 1.86) |
| Low adherence | 17.1 (0.8) | 2.72 (0.89; 4.56) | 1.55 (0.34; 2.76) |
| *TAI scale* |  | *P=0.001* | *P=0.008* |
| Good adherence | 14.3 (0.4) | 0.0 (ref.) | 0.0 (ref.) |
| Intermediate adherence | 15.8 (0.6) | 1.49 (-0.02; 2.99) | 0.68 (-0.31; 1.67) |
| Poor adherence | 17.7 (0.8) | 3.34 (1.52; 5.15) | 1.62 (0.40; 2.83) |

Note: adjusted analyses were performed taking into account sex, age, BMI, smoking history, exacerbations in past year, BODEx index, COTE index, mMRC scale, ERS symptoms score, physical activity and FEV_1_ % predicted.

**Table D.** Crude and adjusted association between adherence and exacerbations in past (N=602, complete information for all variables)

| **Adherence scale** | **Mean (SE)** | **Unadjusted RR (95% CI)** | **Adjusted RR (95% CI)** |
| --- | --- | --- | --- |
| *Morisky scale* |  | *P=0.143* | *P=0.498* |
| High adherence | 1.42 (0.12) | 1.00 (ref.) | 1.00 (ref.) |
| Medium adherence | 1.48 (0.15) | 1.04 (0.81; 1.35) | 0.95 (0.78; 1.15) |
| Low adherence | 1.75 (0.17) | 1.23 (0.96; 1.59) | 1.11 (0.87; 1.39) |
| *TAI scale* |  | *P=0.002* | *P=0.035* |
| Good adherence | 1.29 (0.10) | 1.00 (ref.) | 1.00 (ref.) |
| Intermediate adherence | 1.58 (0.17) | 1.22 (0.94; 1.59) | 1.12 (0.91; 1.38) |
| Poor adherence | 1.95 (0.22) | 1.51 (1.16; 1.97) | 1.24 (1.01; 1.53) |

Note: adjusted analyses were performed taking into account sex, age, BMI, smoking history, CAT score, BODEx index, COTE index, mMRC scale, ERS symptoms score, physical activity and FEV_1_ % predicted.

**Table E.** Correlation matrix between TAI scale and lung function parameters

|  | Pre-BD FEV_1_ | Post-BD FEV_1_ | Pre-BD FVC | Post-BD FVC | TAI scale | TAI scale (erratic non-adherence pattern) | TAI scale (intentionally non-adherence pattern) |
| --- | --- | --- | --- | --- | --- | --- | --- |
| Pre-BD FEV_1_ | 1,00 |  |  |  |  |  |  |
|  |  |  |  |  |  |  |  |
| Post-BD FEV_1_ | **0,98** | 1,00 |  |  |  |  |  |
|  | ***p<0.001*** |  |  |  |  |  |  |
| Pre-BD FVC | **0,91** | **0,91** | 1,00 |  |  |  |  |
|  | ***p<0.001*** | ***p<0.001*** |  |  |  |  |  |
| Post-BD FVC | **0,77** | **0,78** | **0,85** | 1,00 |  |  |  |
|  | ***p<0.001*** | ***p<0.001*** | ***p<0.001*** |  |  |  |  |
| TAI scale | 0,04 | 0,03 | **0,11** | **0,09** | 1,00 |  |  |
|  | *p=0.328* | *p=0.483* | ***p=0.002*** | ***p=0.009*** |  |  |  |
| TAI scale (erratic non adherence pattern) | -0,01 | -0,02 | **0,07** | 0,06 | **0,95** | 1,00 |  |
|  | *p=0.801* | *p=0.586* | ***p=0.044*** | *p=0.085* | ***p<0.001*** |  |  |
| TAI scale (intentionally non-adherence pattern) | **0,09** | **0,08** | **0,15** | **0,12** | **0,89** | **0,70** | 1,00 |
|  | ***p=0.013*** | ***p=0.022*** | ***p<0.001*** | ***p<0.001*** | ***p<0.001*** | ***p<0.001*** |  |
