# Supplementary material for: Adherence to inhaled therapies of COPD patients from seven Latin American countries: The LASSYC study
Source: PLoS One. 2017 Nov 15;12(11):e0186777. doi: 10.1371/journal.pone.0186777 (PMC5687703; doi:10.1371/journal.pone.0186777)
Supplement: S2 File — This is the file containing the study protocol. (PDF) [file pone.0186777.s002.pdf]

AstraZeneca

Non-Interventional Study Protocol

Study Code	D2287R00112
Version	1.0
Date	April 29th, 2016

Latin American Study of 24-hs Symptoms in Chronic Obstructive Pulmonary Disease (COPD) Patients (LASSYC Study)

An observational, multinational, cross sectional primary data collection study to describe symptoms around 24-hs and their relationship with adherence to respiratory treatment, direct costs and PRO in stable COPD patients in Latin America.

AstraZeneca LatAm

1800 Concord Pike

Wilmington DE 189897

United States

TABLE OF CONTENTS PAGE

	TITLE PAGE	1
	TABLE OF CONTENTS	2
	LIST OF ABBREVIATIONS	5
	RESPONSIBLE PARTIES	6
	PROTOCOL SYNOPSIS	8
	AMENDMENT HISTORY	13
	MILESTONES	14
1.	BACKGROUND AND RATIONALE	15
1.1	Background	15
1.2	Rationale	16
2.	OBJECTIVES	17
2.1	Primary Objective(s)	17
2.2	Secondary Objective(s)	17
2.3	Exploratory Objective(s): Not applicable	18
3.	METHODOLOGY	18
3.1	Study Design – General Aspects	18
3.1.1	Data Source(s)	19
3.2	Study Population	21
3.3	Inclusion Criteria	21
3.4	Exclusion Criteria	22
3.5	Participant Follow-up	22
4.	VARIABLES AND EPIDEMIOLOGICAL MEASUREMENTS	22
4.1	Exposures:	24
4.1.1	Definition of Primary Drug Exposure:	24
4.1.2	Definition of Comparison Drug Exposure	24
4.2	Outcomes	24
4.2.1	Primary Endpoint	24
4.2.2	Secondary Endpoints	24
4.3	Other Variables and Covariates.	25

5. STATISTICAL ANALYSIS PLAN 25
5.1 Statistical Methods – General Aspects 25
5.1.1 Primary Objective(s): Calculation of Epidemiological Measure(s) of Interest (e.g. descriptive statistics, hazard ratios, incidence rates, test/retest reliability) 25
5.1.2 Secondary Objective(s): Calculation of Epidemiological Measure(s) of Interest (e.g. hazard ratios, incidence rates, test/retest reliability) 26
5.1.3 Exploratory Objective(s): 26
5.1.4 Bias 26
5.1.5 Strengths and Limitations 26
5.2 Sample Size and Power Calculations 27
6. STUDY CONDUCT AND REGULATORY DETAILS 28
6.1 Data Management 28
6.1.1 Study Flow Chart and Plan 28
6.1.2 Procedures 30
6.1.2.1 Specific procedures for Investigators´ assessment tools 30
a) Disease group (2013 GOLD Recommendation). 30
b) Level of dyspnea (modified MRC -mMRC- dyspnea scale) 32
c) Comorbidities, COTE Index 32
d) BODEx Index 33
e) COPD exacerbations history 34
6.1.2.2 Specific procedures for Patient reported Outcomes questionnaires. 34
a) Day-time, Morning and Night-time Symptoms of COPD questionnaire. (Appendix 2). 35
b) Adherence to respiratory medicines, TAI and MMAS-8 Questionnaires. (Appendix 5) 35
c) COPD Assessment Test (CAT). (Appendix 6) 36
d) International Physical Activity Questionnaire (IPAQ). (Appendix 7) 36
6.1.2.3 Direct Medical Healthcare Resources Utilisation (HRU) 37
6.1.2.4 Current therapy for COPD and others 37
6.1.3 Quality Control 37
6.1.4 Storage and Retention 39
6.2 Protection of Human Subjects 39
6.2.1 Patient Informed Consent 40
6.2.2 Confidentiality of Study/Subject Data 40
6.3 Management and Report of Adverse Events/Adverse Drug Reactions 41
6.3.1 Definition of Adverse Events (AE) 41
6.3.2 Definition of Serious Adverse Events (SAE) 41
6.3.3 Collection of Adverse Events 41
6.3.4 Reporting of Adverse Events 41
6.4 Study Governance and Committees 41
6.5 Communication Plan 43

6.5.1 Publication Plan 43
6.5.2 Compliance with Study Registration and Results Posting Requirements 44
6.5.3 Compliance with Financial Disclosure Requirements 44
6.5.4 Changes to Protocol 44

7. APPENDICES 46

7.1 APPENDIX 1: PATIENT INFORMATION SHEET AND INFORMED CONSENT FORM (ICF) 46

7.2 APPENDIX 2: NIGHT-TIME, MORNING AND DAY-TIME SYMPTOMS OF COPD 49
7.2.1 "Evaluating Respiratory Symptoms in COPD" (E-RS™) Questionnaire. To be used for daytime symptoms of day before to study visit 49
7.2.2 NiSCI Questionnaire. 53
7.2.3 EMSCI Questionnaire. 56

7.3 APPENDIX 3: DISEASE SEVERITY (2013 GOLD DOCUMENT) 59

7.4 APPENDIX 4: MODIFIED MRC DYSPNEA SCALE, mMRC 61

7.5 APPENDIX 5: ADHERENCE QUESTIONNAIRES 62
7.5.1 TAI Questionnaire: 62
7.5.2 The Morisky Scale (MMAS-8) 64

7.6 APPENDIX 6: COPD ASSESSMENT TEST (CAT) 65

7.7 APPENDIX 7: INTERNATIONAL PHYSICAL ACTIVITY QUESTIONNAIRE (IPAQ, August 2002) 66

8. SIGNATURES 68

9. LIST OF REFERENCES 77

LIST OF ABBREVIATIONS

Abbreviation or special term	Explanation
ATS	American Thoracic Association
BTS	British Thoracic Society
CAT	COPD Assessment Test
COPD	Chronic Obstructive Pulmonary Disease
CRA	Clinical Research Associate
CRF	Case Report Form
CRO	Clinical Research Organization
CSM	Clinical Study Manager
EC	Ethics Committee
ERS	European Respiratory Society
FEV1	Forced Expiratory Volume in one second
FVC	Forced Volume Capacity
GCP	Good Clinical Practice
GEP	Good Epidemiological Practices
GOLD	Global Initiative for Chronic Obstructive Lung Disease
HRQoL	Health-Related Quality of Life
HRU	Healthcare Resources Utilisation
ICF	Informed Consent Form
ICH	International Conference on Harmonisation
IPAQ	International Physical Activity Questionnaire
ISPE	International Society for Pharmacoepidemiology
ISPOR	International Society for Pharmacoeconomics and Outcomes Research
MRC	Medical Research Council
SA	Anonymous Society
SAP	Statistical Analysis Plan
SOP	Standard Operating Procedure
UK	United Kingdom
US	United States
WHO	World Health Organization

RESPONSIBLE PARTIES

Name	Professional Title	Role in Study	Affiliation	Email Address
Filip Surmont	AZ LatAm Medical Director	Scientific Lead. Regional Project Team.	Medical Director AZ LatAm. AstraZeneca, Wilmington USA	Filip.surmont@astrazeneca.com
Valentina Di Boscio	AZ LatAm Medical Respiratory Lead	Project Lead. Protocol writing. Regional Project Team	Respiratory Medical Lead AZ LatAm. AstraZeneca, Buenos Aires Argentina	Valentina.diboscio@astrazeneca.com
Jaime Solorzano	AZ Camcar Market Access Lead	Health Economics Research Manager. Regional Project Team	Market Access Lead AZ Camcar. AstraZeneca, Costa Rica	Jaime.solorzano@astrazeneca.com
Enrique Gaubeca	Klixar General Manager	Clinical Research Organization (CRO) Operational Lead for Argentina, Chile, Colombia, Mexico and Uruguay	Klixar Buenos Aires, Argentina	Enrique.gaubeca@klixar.com
Florencia Licastro	Klixar Clinical Operations Manager	Clinical Research Organization (CRO) Operational Lead for Argentina, Chile, Colombia, Mexico and Uruguay	Klixar Buenos Aires, Argentina	Florencia.licastro@klixar.com
Marioli Castillo	Global Medical Logistic (GML) Executive Director	Clinical Research Organization (CRO) Project Director for Costa Rica and Guatemala	GML Guatemala, Guatemala	Marioli.castillo@gmedical.net
Hellen Sancho	Global Medical Logistic (GML) Project Manager	CRO Operational Lead for Costa Rica and Guatemala	GML Guatemala, Guatemala	Hellen.sancho@gmedical.net

**Name**	**Professional Title**	**Role in Study**	**Affiliation**	**Email Address**
Ana Baptista Menezes	Epidemiologist and Statistician	Epidemiologist Statistician provider	Professor of Respiratory Medicine and Epidemiology, Federal University of Pelotas, Brazil Rua Marechal Deodoro 1160, Pelotas, RS, Brazil	Anamene.epi@gmail.com

PROTOCOL SYNOPSIS

Latin American Study of 24-hs Symptoms in Chronic Obstructive Pulmonary Disease Patients (LASSYC Study)

An observational, multinational, cross sectional primary data collection study to describe symptoms around 24-hs and their relationship with adherence to respiratory treatment, direct costs and PRO in stable COPD patients in Latin America.

Background/Rationale:

No previous studies have evaluated the frequency and severity of chronic obstructive pulmonary disease (COPD) symptoms over a period of 24 hours (night-time, morning and day-time symptoms) in stable COPD patients seen in clinical practice in Latin America. COPD is a common disorder seen by primary care physicians and one of the most common diseases referred and diagnosed by pulmonologists. According to studies, symptoms of COPD can have a substantial impact on patients' quality of daily life and present a considerable degree of variation for the same degree of airflow limitation.

This study aims to learn more about the burden of symptoms in the real-world population of COPD patients in Latin America. With the real life data coming from this study, it will be possible to describe 24-hour COPD symptoms in Latin America and their impact on patients' quality of life and other PRO, the relationship with patients´ behaviour regarding adherence to respiratory medication and burden of COPD symptoms in terms of the impact on health economics.

In the present study, we will assess and characterize COPD symptoms over a period of 24 hours, by collecting information about the respiratory symptoms experienced at different times of the day and night-time in patients with stable COPD under real clinical practice conditions. In addition, we will evaluate the correlation between each of these symptoms and the GLOD classification, adherence to respiratory treatment, level of dyspnea, disease severity, comorbidities and physical activity. Finally, we will assess the relationship between 24h symptoms and direct cost related to treatment and HRU in previous year to assess the burden of COPD symptoms.

Objectives:

Primary Objective: To describe prevalence, severity and interrelationship of early morning, day and night-time symptoms in patients with stable Chronic Obstructive Pulmonary Disease (COPD) in LatAm.

Secondary Objectives:

1. To evaluate the relationship between early morning, day and night-time symptoms and:
   - Adherence to respiratory treatment
   - Disease GOLD 2013 classification, severity (BODEX), level of dyspnea (mMRC), comorbidities (COTE)
   - Exacerbation history in previous 12 months
   - PRO: HRQoL(CAT) and physical activity (IPAQ)
   - Direct Cost of the disease (Healthcare Resource Use – HRU – in past 12 months and medication costs in last 2 months without any change)

2. To describe treatment in Latin America COPD patients according therapeutic class and modality (rescue vs maintenance)

No formal hypotheses testing will be performed.

Methods:

Study design:

This is a multi-country, multicentre, observational prospective data collection cross sectional study of patients with stable COPD in Latin America.

Data Source(s):

Site staff will retrospectively collect the necessary information from the patient's medical record to determine eligibility. Consecutively, patients with diagnosis and stable COPD will be screened and, if eligible, consented and enrolled. There will be only one study visit. For each patient, the physician will collect the following data at visit (from medical records or interviewing in study visit): social demographics, health insurance system, lifestyle, smoking history, comorbidities, level of dyspnea, disease severity, COPD prescribed treatments including therapeutic class, device, modality (rescue/maintenance) and posology during last past 2 months, exacerbations history and healthcare resources utilisation during last 12 months.

In addition, the patient will be asked to provide data on disease-related symptomatology assessed during 24 hs day (early morning, daytime and night time symptoms), adherence to inhalers, health-related quality of life (HRQoL) and physical activity.

Study Population:

Male and female ambulatory patients aged over 40 years diagnosed with stable COPD according 2013 GOLD criteria.

Inclusion criteria

Patients will only be included in the study if they meet all of the following criteria:

1. Male or female patients aged 40 years or older.
2. Patient has diagnosis of COPD for 1 year or more.
3. Patient has at least one spirometry with COPD criteria, fixed ratio <0.70 post BD, in previous 12 months
4. Patient is a current smoker or an ex-smoker with a smoking history of ≥ 10 pack-years.
5. Stable patients, as stated in medical records or patient reports during visit, defined as: without exacerbation treatment at study visit neither in the previous 2 months, and without changes in maintenance COPD treatment regimen over the preceding 2 months (avoid first consult patient)
6. Patients must be able and willing to read and comprehend written instructions, and comprehend and complete the questionnaires required by the protocol
7. After full explanation, patients must have signed an informed consent document indicating that they understand the purpose of and the procedures required for the study and are willing to participate in the study.

Exclusion criteria

Patients who meet any of the following criteria will not be eligible to participate in the study:

1. Patient has diagnosis of sleep apnea syndrome or other chronic respiratory disease different from chronic obstructive diseases.
2. An acute or chronic condition that, in the investigator's opinion, would limit the patient's ability to complete questionnaires or participate in this study
3. Patient participating in any other medical investigation currently at study visit.

Exposure(s):

The LASSYC study is an observational cross sectional study which does not involve or study a specific medicinal product. Information about exposure to treatments as part of routine care will be collected (frequency, treatment, duration).

Outcome(s):

Primary endpoints will include:

- Frequency of night-time symptoms related to COPD
- Mean severity of night-time symptoms related to COPD.
- Frequency of day-time symptoms related to COPD.
- Mean severity of day-time symptoms related to COPD.
- Frequency of early morning symptoms related to COPD.

- Mean severity of early morning symptoms related to COPD.
- Interrelationship between early mornings, day and night-time symptoms.

Secondary endpoints will include:

- Relationship between early morning, day and night-time symptoms and adherence to respiratory medication
- Relationship between early morning, day and night-time symptoms and disease classification (GOLD 2013)
- Relationship between early morning, day and night-time symptoms and disease severity and prognostic assessment (BODEX).
- Relationship between early morning, day and night-time symptoms and level of dyspnea (mMRC).
- Relationship between early morning, day and night-time symptoms and HRQoL (CAT).
- Relationship between early morning, day and night-time symptoms and presence of comorbidities (COTE).
- Relationship between early morning, day and night-time symptoms and history of exacerbations in last 12 months
- Relationship between early morning, day and night-time symptoms and physical activity (IPAQ).
- Relationship between early morning, day and night-time symptoms and direct costs related to medication use and HRU in last 12 months

Sample Size Estimations:

A sample size of 860 patients offers a maximum margin of error (minimum precision) of 4% for estimating the percentage of patients within each category of the primary endpoint (frequency of early morning, day and night-time COPD symptoms), considering maximum indetermination (p=40%) and a confidence level of 95%. Considering that approximately 5% of patients will not be evaluable (missing data or major protocol violation), the final sample size will be 900 patients. No specific patient number from each participating country as analysis will be done for the complete LatAm cluster, although 100 to 130 patients are expected for each country.

Statistical Analysis:

The statistical analyses will be fully described in Statistical Analysis Plan (SAP) as appropriate**.**

After data collection, data will be summarized for the population overall for the primary and secondary objectives.

For direct costs the aim is to make per country descriptive analysis of the use of resources (prescribed treatment during past 2 months, medical consultations and exacerbations during previous year) and the estimation of direct medical cost per patient in each country.

Descriptive analyses of variables collected will be performed. For the secondary objectives, relationships between variables will be assessed using appropriate statistical tests according to the nature of the variables. No formal hypotheses testing will be performed.

All the health economics data related to medication use will be capture in the monthly expenditure on drugs determined by micro-costing techniques, considering the average price of the presentations available on the market in each country, without adjustments.

Cost of the disease per country related to HRU will be based extrapolating local values of healthcare resources. The values estimated in local currency, will be converted to international dollars (USD$).

AMENDMENT HISTORY

Date	Brief description of change	Administrative Change / Amendment / New Protocol Version.
<< >>	<< >><br>N/A	

MILESTONES

Date	Milestone
November 2015	Development of Study Concept Sheet
March 2016	Final Protocol
April 2016	Study Start Up: contracts in place, regulatory submissions, initiation visits
July 2016	FSI
September 2016	LSI
October 2016	Database Lock & Data Extraction
October 2016	Development of Analytic Datasets
December 2016	Final Report
Q1 2017	First Main Manuscript

1. BACKGROUND AND RATIONALE

1.1 Background

Chronic obstructive pulmonary disease (COPD) is a preventable and treatable disease with some significant extrapulmonary effects that may contribute to the severity in individual patients. Its pulmonary component is characterized by airflow limitation that is not fully reversible. The airflow limitation is usually progressive and associated with an abnormal inflammatory response of the lung to noxious particles or gases (1). Cigarette smoking causes 80%-90% of all cases of COPD. The structural changes in the airways that are characteristic of COPD result from repeated injury and repair and by bronchoconstriction, which is one of the key targets for pharmacologic interventions.

This disease affects globally around 15% of all adults older than 40 years, resulting in pulmonary and extrapulmonary comorbidities and significant mortality (1,2). The worldwide prevalence in people aged over 40 years is 10.1% overall, 11.8% for men, and 8.5% for women, according to the BOLD Study (3). It is the fourth leading cause of mortality in the world and its prevalence is projected to increase in the coming years due to continued exposure to COPD risk factors (1). Two Latin-American epidemiological studies, the PLATINO Project 4 and PREPOCOL 5, have provided information on the prevalence of COPD in this region. PLATINO is a study on prevalence of COPD in individuals ≥40 years, carried out in five Latin American cities: Mexico City (Mexico), San Pablo (Brazil), Montevideo (Uruguay), Santiago de Chile (Chile), and Caracas (Venezuela). Using as diagnostic criteria post-BD $FEV_1/FVC<0.70$, the overall prevalence of COPD in PLATINO is 14.3% (ranging from 7.8% in Mexico City to 19.7% in Montevideo)(4,6). Using the criteria of the lower limit of normal (LLN) of the post-BD FEV_1/FVC ratio, the global COPD prevalence in PLATINO is 11.7%. This is even lower (9.5%) when using the post-BD $FEV_1/VEF_6<LLN$ ratio (7). PREPOCOL (5) assessed the prevalence in five cities in Colombia and reported an overall prevalence of 8.9% (from 6.2% in Barranquilla to 13,5% in Medellin).

Symptoms of COPD – including progressive dyspnea, chronic cough, excessive sputum production and decreased exercise tolerance – can impact considerably on patients' daily activities and quality of life (1,8,9). COPD symptoms have been reported to be worse at night and in the early morning (10,11), which may be reflected in disturbed sleep and limitations on morning activities.

Moreover, the health-related quality of life (HRQoL) is being recognized as an important outcome when evaluating patients with COPD and diverse studies have demonstrated that those patients in more severe disease stages presented poorer HRQoL and also it varied greatly within each stage of disease severity (12).

From the patient´s point of view, dyspnea is one of the major symptoms that affects their quality of life (13). However, dyspnea varies considerably for the same degree of airflow limitation.

It is reported that poor patient's adherence to inhaled medication is shared by COPD and asthma patients (14). Non-adherence to inhaled therapy is associated with poor symptom control, higher healthcare utilization and costs as well as decreases in health-related quality of life (12-14). In practice guidelines of the Global Initiative for Asthma Management [GINA) (15) and Global Initiative for Chronic Obstructive Lung Disease (GOLD) (1)] is now specifically recommended evaluation and follow-up of the patient's adherence to inhaler devices.

Previous studies (16, 17, 18, 19) have shown that physical activity in COPD is associated with a better quality of life and less morbidity and mortality. Besides, comorbid conditions such as cardiovascular disease, anxiety and depressive disorders, lung cancer and osteoporosis are often observed in COPD patients and are likely to affect COPD outcomes (20). Sleep disturbance is also common in COPD patients. In epidemiologic studies, more than 50% of patients with COPD complained of difficulty maintaining and initiating sleep, and 25% complained of excessive daytime sleepiness (21). The COTE Index establishes the relationship between these main comorbidities and the risk of death over a median of 51 months. This is a simple disease specific´s comorbidity index that helps to assess mortality risk in patients with COPD (22).

It has been recently published the ASSESS Study (23) in a cohort of 727 European patients. This observational study assessed the prevalence, severity and relationship between nighttime, early morning and daytime COPD symptoms and explored the relationship between 24-hour symptoms and other patient-reported outcomes. It was shown that more than half of patients experienced COPD symptoms throughout the whole 24-hour day and there was a significant relationship between them and worse patient-reported outcomes, suggesting that improving 24-hour symptoms should be an important consideration in the management of COPD

COPD is associated with a substantial economic burden due to direct healthcare costs e.g. unplanned medical visits, hospitalization for exacerbations (1,24). More frequent exacerbations are associated with: more frequent/longer hospital stays; increased mortality risk; reduced quality of life (25) also indirect/societal costs due to loss of productivity and premature death in patients with COPD further add to the economic burden of this disease (1,24-26).

1.2 Rationale

No previous studies have evaluated the frequency and severity of COPD symptoms over a period of 24 hours (night-time, morning and daytime symptoms) in stable COPD patients seen in clinical practice in Latin America. COPD is a common disorder seen by primary care physicians and one of the most common diseases referred and diagnosed by pulmonologists. According to studies, symptoms of COPD can have a substantial impact on patients' quality of daily life and present a considerable degree of variation for the same degree of airflow limitation.

This study aims to learn more about the burden of symptoms in the real-world population of COPD patients in Latin America. With the real life data coming from this study, it will be possible to describe 24-hour COPD symptoms in Latin American and their impact on patients' quality of life and other PRO, the relationship with patients´ behaviour regarding adherence to respiratory medication and burden of COPD symptoms in terms of the impact on health economics.

In the present study, we will assess and characterize COPD symptoms over a period of 24 hours, by collecting information about the respiratory symptoms experienced at different times of the day and night-time in patients with stable COPD under real clinical practice conditions. In addition, we will evaluate the correlation between each of these symptoms and the 2013 GOLD classification, adherence to respiratory treatment, level of dyspnea, disease severity, comorbidities and physical activity. Finally, we will assess the relationship between 24h symptoms and direct cost related to treatment and HRU in previous year to assess the burden of COPD symptoms.

2. OBJECTIVES

The purpose of the study is to characterise and determine the prevalence and severity of early morning, day and night-time symptoms and to evaluate their correlation between each other and with disease severity, adherence to inhalers, direct costs and PROs in patients with stable Chronic Obstructive Pulmonary Disease (COPD) in Latin America.

No formal hypothesis will be tested.

2.1 Primary Objective(s)

- To describe prevalence, severity and interrelationship of early morning, day and night-time symptoms in patients with stable COPD in Latin America.

2.2 Secondary Objective(s)

1. To evaluate the relationship between early morning, day and night-time symptoms and:
    - Adherence to respiratory treatment
    - Disease GOLD 2013 classification, severity and prognostic assessment (BODEx), level of dyspnea (mMRC), comorbidities (COTE)
    - Exacerbation history in previous 12 months
    - PRO: HRQoL(CAT) and physical activity (IPAQ)
    - Direct Cost of the disease (Healthcare Resource Use – HRU – in past 12 months and medication costs in last 2 months without any change).

2. To describe treatment in Latin America COPD patients according therapeutic class and modality (rescue vs maintenance)

2.3 Exploratory Objective(s): Not applicable

3. METHODOLOGY

3.1 Study Design – General Aspects

The study is a multi-centre, multi country, observational prospective cross sectional study to determine the prevalence and severity of early morning, day and night-time symptoms and to evaluate their correlation between each other and with disease severity, adherence to respiratory medications, direct costs and PROs in patients with stable COPD in Latin America.

Approximately 900 patients will be enrolled distributed among convenient sites in seven-selected countries: Argentina, Chile, Colombia, Costa Rica, Guatemala, México and Uruguay. It is expected that each country recruit 100 – 130 patients and each site will recruit 10-15 patients that meet the eligibility criteria of stable COPD. The recruitment will be competitive inside each country after the expected site recruitment time of one month, up to total of three months recruitment period.

The study team will identify suitable ambulatory pulmonology sites (i.e., those whose databases will have information on diagnostic procedures, etc) to ensure an adequate sample of both physicians and patients.

Pseudo anonymised data will be collected over the course of the study. Patients will be identified consecutively and, if they meet eligibility criteria and provide consent will be enrolled.

Patients should have COPD diagnosis for one year ago or more. At study visit, selected patients will be asked to complete questionnaires for early morning, day and night-time symptoms, adherence to inhalers, HRQoL, and physical activity.

The investigator will be requested to complete study electronic case report forms (eCRFs) recording specified information (demographics, lifestyle data, medical history and comorbidities, level of dyspnea, disease severity according 2013 GOLD guidelines, COPD exacerbations and healthcare resources utilisation during last year, and COPD prescribed treatments in last 2 months). No additional mandated interventions on top of routinely performed physician visits, examinations or treatments will be required.

Any procedure ordered by the physician during this study will be one that is appropriate to the routine clinical care delivered to the patient at the discretion of the participating physician.

At the end of the study, a descriptive and analytical analysis will be performed. A separate Statistical Analysis Plan (SAP) will be written to specify the analyses in detail.

The study will be conducted in accordance with the guidelines set forth by the International Society for Pharmacoepidemiology (ISPE) [[27]] and the International Society for

Pharmacoeconomics and Outcomes Research (ISPOR) [28] for the conduct of burden of disease studies.

Direct cost of the disease per country will be estimated based on the cohort analysed extrapolating with the local prevalence of COPD and Healthcare resources.

All the health economics date will be capture in the monthly expenditure on drugs determined by micro-costing techniques, considering the average price of the presentations available on the market in each country, without adjustments. The values estimated in local currency, will be converted to international dollars ($USD).

The study will be complete when the last subject completes the study visit.

3.1.1 Data Source(s)

Site staff will retrospectively collect the necessary information from the patient's medical record to determine eligibility. Consecutively, patients with a formal spirometric diagnosis and stable COPD will be screened and, if eligible, consented and enrolled.

For each patient, the physician will collect data from medical records or interviewing in study visit: social demographics, health insurance system, lifestyle, smoking history, comorbidities, level of dyspnea, disease severity, COPD prescribed treatments including therapeutic class, device, modality (rescue/maintenance) and posology during last past 2 months, exacerbations history and healthcare resources utilisation during last 12 months.

In addition, the patient will be asked to provide data on disease-related symptomatology assessed during 24-hs day (early morning, daytime and night time symptoms), adherence to respiratory medicines, health-related quality of life (HRQoL) and physical activity.

No additional mandated interventions on top of routinely performed physician visits, examinations or treatments will be required.

The US National Heart, Lung, and Blood Institute and the World Health Organization to bring more attention to COPD, its management, and its prevention (1) formed the Global Initiative for Chronic Obstructive Lung Disease (GOLD) in 1998. Spirometry is essential for diagnosis and provides a useful description of the severity of pathological changes in COPD. Moreover, FEV_1 and FVC measurements are highly reproducible if performed adequately. Current GOLD recommendations classify the disease into four Groups (A to D) according symptom´s impact (using specific cut points for CAT, mMRC or CCQ) and disease´s risk (using specific spirometric or previous exacerbations cut points).

The Medical Research Council (MRC) dyspnea scale ([29]) has been in use for many years for grading the effect of breathlessness on daily activities. This scale actually measures perceived respiratory disability, the WHO definition of disability being "any restriction or lack of ability to perform an activity in the manner or within the range considered normal for a human being". The MRC dyspnoea scale is simple to administer as it allows the physician to indicate

the extent to which the breathlessness affects the mobility of the patient. Moreover, the MRC dyspnoea scale is a valid method of categorising patients with COPD in terms of their disability that could be used to complement FEV_1 in the classification of COPD severity. The method has been widely used in the past ([30],[31]).

Multidimensional indices such as the BODEx index ([32]) (body mass index, airflow obstruction, dyspnea, and frequency of severe exacerbations) have demonstrated excellent prognostic value. As the 6-minute walking test considered in the BODE index ([33]) is not performed routinely it and may be substituted by exacerbations providing similar prognostic properties (29) simplifying without loss of predictive capacity. These index integrate the principal prognostic determinants in COPD patients. BODE Index (30) showed to be more effective than FEV_1 as a prognostic variable and unlike the latter, it contains a number of dimensions that can be modified – such as dyspnea, or Body Mass Index (BMI).

The COTE Index (22) establishes the relationship between these main comorbidities and the risk of death over a median of 51 months. The prevalence and mortality risk are expressed through a quantitative risk stratification comorbidity tool.

The effect of the COTE index on the risk of death varied over time. There was minimal evidence that an increased COTE index was associated with an increase in the hazard of death in patients with COPD followed for less than 18 months (HR, 1.04; 95% confidence interval [CI], 0.98–1.10; P ¼ 0.18). In contrast, an increased COTE index was associated with death in patients with COPD followed for greater than or equal to 18 months (HR, 1.16; 95% CI, 1.13–1.19; p= 0.001).

The COPD Assessment Test (CAT) was developed by a multidisciplinary group of international experts, commissioned, and funded by GlaxoSmithKline (34). The CAT is a validated, short and simple patient completed questionnaire that has been developed for use in routine clinical practice to measure the health status of patients with COPD. Despite the small number of component items, it covers a broad range of effects of COPD on patients' health.

The International Physical Activity Questionnaire (IPAQ) will be used in its short (4 generic items) versions for self-administered use (35). In the present study, the short form will be used because it is more appropriate in an observational study. IPAQ short form is an instrument designed primarily for population surveillance of physical activity among adults. It has been developed and tested for use in adults (age range of 15-69 years). The questionnaire is the most feasible instrument for measuring physical activity in large groups or populations. There are validated versions available in more than 10 languages and has been widely used.

The Test of the Adherence to Inhalers (TAI) has been developed and validated to identify adherence and nonadherence behaviour patterns to inhaler devices in patients with COPD and asthma (14). It is a reliable, homogeneous, one dimensional questionnaire to classify from a clinical perspective the barriers related to the use of inhalers in asthma and COPD. Includes 12 items, the first 10 items (patient domain) are self-administered and scored from 1 to 5 (where 1 = worst possible score and 5 = best possible score). The final two items are completed by the healthcare professionals and scored as 1 or 2 (where 1 = bad and 2 = good).

These items were designed to identify unwitting non-adherent behaviour (failure in understanding medication use, dosage or inhalation technique). Total score ranges between 10 and 50 for the 10-items TAI, and between 12 and 54 for the 12-items TAI Adherence is rated as good, intermediate or poor in the presence of 50, 46 to 49 and < 45 points in the 10-items questionnaire (14).

The eight-item Morisky Medication Adherence Scale (MMAS-8) (36) is a structured self-report measure of medication-taking behaviour that has been widely used in various cultures. The Morisky scale (33) was developed initially to assess adherence to medication use in patients with essential hypertension but has subsequently been used in other studies of asthma medication compliance (37 38). Items 1 to 7 are Yes/No questions; for which Yes is scored as 0 and No as 1 point; except for question 5 which answer Yes is scored as 1 and No as 0 point. Item 8 is a ranked answer question type Likert Scale from 0-4. Patient adherence is classified into 3 categories based on responses to the Morisky scale (33) These included high – medium - low adherence.

electronic Case Report Form (eCRF)

The eCRF will be used to collect all variables about patients through their physicians or study personnel. The eCRF guides the investigator through the registration of patients, collection of data and management of the study. Since it will be critical to be able to analyze data collected across multiple countries, the eCRF will be used to ensure a consistent core data collection for each physician involved in the LASSYC study. The eCRF will be accessed through secure AstraZeneca web-based portal accordingly to different users profiles.

3.2 Study Population

Approximately 900 stable COPD patients will be enrolled distributed among the seven selected study countries: Argentina, Chile, Colombia, Costa Rica, Guatemala, México and Uruguay. It is expected that each country recruit 100 – 130 patients and each site will recruit 10-15 patients that meet the eligibility criteria of stable COPD.

The recruitment will be competitive inside each country after the expected site recruitment time of one month, up to total of three months recruitment period.

The study teamr will identify suitable ambulatory pulmonology sites (i.e., those whose databases will have information on diagnostic procedures, etc) to ensure an adequate sample of both physicians and patients. Centers/sites will be distributed as convenience among countries, located in capital or inner cities according local best consideration and they should be outpatient clinics of specialists investigators (pulmonologists) experienced in GCP.

3.3 Inclusion Criteria

Patients will only be included in the study if they meet all of the following criteria:

1. Male or female patients aged 40 years or older.
2. Patient has diagnosis of COPD for 1 year or more.

3. Patient has at least one spirometry with COPD criteria, fixed ratio <0.70 post BD, in previous 12 months
4. Patient is a current smoker or an ex-smoker with a smoking history of ≥ 10 pack-years.
5. Stable patients, as stated in medical records or patient reports during visit, defined as: without exacerbation treatment at study visit neither in the previous 2 months, and without changes in maintenance COPD treatment regimen over the preceding 2 months (avoid first consult patient)
6. Patients must be able and willing to read and comprehend written instructions, and comprehend and complete the questionnaires required by the protocol
7. After full explanation, patients must have signed an informed consent document indicating that they understand the purpose of and the procedures required for the study and are willing to participate in the study.

3.4 Exclusion Criteria

Patients who meet any of the following criteria will not be eligible to participate in the study:

1. Patient has diagnosis of sleep apnea syndrome or other chronic respiratory disease different from chronic obstructive diseases.
2. An acute or chronic condition that, in the investigator's opinion, would limit the patient's ability to complete questionnaires or participate in this study
3. Patient participating in any other medical investigation currently at study visit.

3.5 Participant Follow-up

Eligible patients will be enrolled in the study at the time they routinely visit their physician and consent to participate in the study. As this is a cross sectional one visit study, patients will be asked to complete study assessments same day of routinely visit.

Patients may withdraw consent at any time and no further data will be collected for a patient who withdraws consent. Patients will not be replaced.

4. VARIABLES AND EPIDEMIOLOGICAL MEASUREMENTS

The variables will be collected during routine clinical visits. The LASSYC study is a cross sectional study, it does not involve or study a specific medicinal product and therefore will fall under the scope of the regulations and guidelines applicable to observational studies. The majority of variables to be collected are part of routine general health assessment or specific guidelines for the management of COPD patients, and are/should be routinely collected, according to international respiratory care guidelines and/or recommended medical practice guidelines. All variables can be collected through non-invasive procedures.

The following list covers the types of variables for which collection is envisioned.

Variables to collect by the investigator:

- Socio-demographic: country, sex, age.
- Anthropometrics: weight, height.
- Socio-economic: educational level, employment status and healthcare type of system.
- Lifestyle: smoking status/history, alcohol consumption, physical exercise.
- Medical history including date of COPD diagnosis and comorbidities:
    - previous vaccination (pneumonia and flue),
    - previous diagnosis of Asthma
    - Lung, esophageal, pancreatic or breast cancer
    - Diabetes Mellitus with neuropathy
    - Hypertension, Atrial fibrillation, Cerebrovascular disease, Peripheral arterial disease and Coronary heart disease
    - Pulmonary fibrosis
    - Anxiety
    - Liver cirrhosis
    - Gastric/duodenal ulcers
    - Osteoporosis
- Level of dyspnea, evaluated by the modified MRC dyspnea scale (0-4).
- Pulmonary function:
    - date of last spirometry available within previous year
    - FVC ml and % pre and post bronchodilator
    - FEV_1 ml and % pre and post bronchodilator
    - FEV_1/FVC pre and post bronchodilator
- COPD exacerbations in the last year: number and attending setting (ambulatory, emergency room, hospitalized or intensive care unit).
- Healthcare resources utilisation during last year: number of COPD related visits and type of doctor (general practitioner/family doctor or pulmonologist/internist).
- Current medications for COPD if any: drug name, therapeutic class, daily doses, modality -rescue/maintenance.
- Disease group classification according 2013 GOLD recommendations (impact of symptoms, frequency of exacerbations previous year and airflow limitation)
- Disease severity and prognostic assessment according BODEX

Patients´ variables (PRO collected at visit study):

- Day symptoms using “Evaluating Respiratory Symptoms in COPD” E-RS™ (formerly known as EXACT-RS, version 2009, copyright 2013).
- Night symptoms using NiSCI 11-item questionnaire version 2014
- Early morning symptoms using EMSCI 10-item questionnaire version 2014
- HRQoL assessed with the COPD Assessment Test (CAT, version 2009).
- Adherence to respiratory medication (TAI version available on line April 2016 and MMAS-8 version 2008 licensed to AZ May 2016)
- Physical activity assessed with the IPAQ (version 2003)

4.1 Exposures:

The LASSYC study is an observational cross sectional, which does not involve or study a specific medicinal product. Information about exposure to treatments as part of routine care will be collected (frequency, treatment and duration).

4.1.1 Definition of Primary Drug Exposure:

Not applicable

4.1.2 Definition of Comparison Drug Exposure

Not applicable

4.2 Outcomes

4.2.1 Primary Endpoint

The primary endpoints are the following, reported for the study population as a whole:

- Frequency of night-time symptoms related to COPD
- Mean severity of night-time symptoms related to COPD.
- Frequency of day-time symptoms related to COPD.
- Mean severity of day-time symptoms related to COPD.
- Frequency of early morning symptoms related to COPD.
- Mean severity of early morning symptoms related to COPD.
- Interrelationship between early mornings, day and night-time symptoms.

4.2.2 Secondary Endpoints

The secondary endpoints are the following, reported for the study population as a whole

- Relationship between early morning, day and night-time symptoms and adherence to respiratory medication
- Relationship between early morning, day and night-time symptoms and disease classification (GOLD 2013)
- Relationship between early morning, day and night-time symptoms and disease severity and prognostic assessment (BODEx).
- Relationship between early morning, day and night-time symptoms and level of dyspnea (mMRC).
- Relationship between early morning, day and night-time symptoms and HRQoL (CAT).
- Relationship between early morning, day and night-time symptoms and presence of comorbidities (COTE).
- Relationship between early morning, day and night-time symptoms and history of exacerbations in last 12 months

4.3 Other Variables and Covariates.

Not applicable.

5. STATISTICAL ANALYSIS PLAN

5.1 Statistical Methods – General Aspects

A fully specified Statistical Analysis Plan (SAP) will be prepared by the awarded statistical provider- before the data base lock. The Study team of AstraZeneca will review this SAP. The contents of the SAP will follow AstraZeneca´s internal Standard Operating Procedure, containing the study objective, responsibilities, type of primary analysis, clear specification of all primary and secondary variables, full and detailed description of the statistical methods for data analysis and shell samples of all results presentation formats and data listings.

All tabulations, figures and listings will be produced using the SAS System (version 9.1.3, at least) - They will be presented in Word documents without any manual editing, i.e. they will appear unmodified as programmed by means of the statistical package. A complete set of raw data listings will be included as an appendix to the Final Study Report.

All SAS datasets, statistical programs and macros used to analyse this study will be provided to AstraZeneca LatAm in a suitable format, just to be able to reproduce at sponsor's site all the reported results for this study.

Statistical analyses will be of explorative and descriptive nature. The study is not designed to confirm (or refute) pre-defined hypotheses.

All variables will be analysed descriptively with appropriate statistical methods: categorical variables by frequency tables (absolute and relative frequencies) and continuous variables by sample statistics (i.e., mean, standard deviation, minimum, median, quartiles and maximum).

5.1.1 Primary Objective(s): Calculation of Epidemiological Measure(s) of Interest (e.g. descriptive statistics, hazard ratios, incidence rates, test/retest reliability)

All analyses will be performed for full analysis set (FAS) that will comprise all patients who fulfil all the eligibility criteria and for whom the primary variables are completed considering the sample as a whole (no country analysis). Whenever reasonable, subgroup analyses will be performed on variables such as age, gender, comorbidities, disease severity, etc.

The analyses will be performed using the available data only method.

All background data such as patient demographics (sex, age), anthropometrics (weight, height), socio-economic information (educational level, employment status), and COPD medication will be described by presenting frequency distributions and/or basic summary statistics. Statistical analyses of demographic and disease characteristics, early morning, night

and day-time symptoms, disease severity, dyspnea, adherence to respiratory medicine, exacerbations during last year, health resource utilisation, HRQoL and physical activity data will be performed.

Prevalence and mean severity of early morning, day and night-time symptoms will be analysed descriptively with appropriate statistical methods. Prevalence and severity of night-time, early morning and day-time symptoms will be assessed based on the symptoms questionnaires respectively.

These analyses will be further described in the SAP.

5.1.2 Secondary Objective(s): Calculation of Epidemiological Measure(s) of Interest (e.g. hazard ratios, incidence rates, test/retest reliability)

For the secondary objectives, relationships between variables will be assessed using appropriate statistical tests according to the nature of the variables.

The relationships between early morning, day and night-time symptoms and adherence to respiratory medication and other COPD disease´s features (level of dyspnea, disease severity, and COPD exacerbations), HRQoL, comorbidities, HRU and impact on physical activities will be evaluated with appropriate statistical methods.

These analyses will be further described in the SAP.

5.1.3 Exploratory Objective(s):

Not applicable

5.1.4 Bias

There might be some gaps and bias in the data due to the observational nature of this study. Sampling process (sites selection and patient recruitment) and sample size might be the more relevant related with the nature of this methodology and are discussed as study limitations.

5.1.5 Strengths and Limitations

LASSYC is expected to enrol approximately 900 patients with diagnosis of stable COPD by a set of physicians from community and hospital outpatient settings, from seven countries within Latin America with a minimum of inclusion and exclusion criteria. This condition would allow to build observational evidence coming from a diverse sample of patients and treatment patterns in Latin America.

The primary data collection tool will be an eCRF. The main advantage of using a primary data source is to maintain the consistency of a single approach for prospective data thus ensuring comparability of data across widely spread out geographies.

In addition, the collection of data directly from the patients through the PRO questionnaires provides a unique perspective and enables the collection of symptom assessments, HRQoL outcomes, and other health information that may not be recorded by their physicians.

Despite the above-mentioned strengths of the study approach, this study is subject to limitations as all studies are.

Selection bias:

- Sites selection:
    - Convenient sample of sites according local study team judgment. External validity could be bias as patients will come from selected clinics.
    - Recruitment through specialist's clinical practice could bias the study population towards those more symptomatic patients. Limited analysis per each GOLD group would be possible in such selected sample .It is reported that almost 70% of patients attended by pneumologist are GOLD groups B and D.
    - Recruitment through clinical practice could bias the study population towards those with more frequent health care utilisation, hence more severe patients.
- Sample size:
    - Sample size guarantee for primary endpoint, hence secondary analysis could be limited.
    - Analysis will be based on total regional sample size, specific countries analysis won´t be possible

  In order to minimise selection bias, eligible patients will be enrolled consecutively at each site with a maximum quota per site. National coordinators shall play an important role in order to ensure PI´s required profile (chest physicians/pulmonary specialists with Good Clinical Practice –GCP- expertise)

  The handling of missing data will be detailed in the SAP.

Information bias: as in clinical practice there is potential for misclassification in most data elements (e.g. diagnosis, severity, level of control assessment, exacerbations, etc.). Patients selected from specialists sites attempt to minimise disease diagnosis.

Methodological bias: the cross sectional nature of this study does not allow analysis on patient´s treatment related with symptoms nor any other variable.

5.2 Sample Size and Power Calculations

A sample size of 860 patients offers a maximum margin of error (minimum precision) of 4% for estimating the percentage of patients within each category of the primary endpoint (frequency of early morning, day and night-time COPD symptoms), considering maximum indetermination (p=40%) and a confidence level of 95%. Considering that approximately 5% of patients will not be evaluable (missing data or major protocol violation), the final sample size will be 900 patients. No specific patient number from each participating country as

analysis will be done for the complete LatAm cluster, although 100 to 130 patients are expected for each country.

6. STUDY CONDUCT AND REGULATORY DETAILS

6.1 Data Management

AstraZeneca will develop Data Management Plan (DMP) and a website database platform for the purpose of this study. DMP will be created before data collection begins and will describe all functions, processes, and specifications for data collection, cleaning and validation.

The eCRFs will include programmable edits to obtain immediate feedback if data are missing, out of range, illogical or potentially erroneous.

Investigators directly through eCRF will perform data entry, hence each of them would be provided with system credentials. Regional and Local Study team, National Coordinators and investigators will be system users allowing different functions on database according individual profiles.

Printed template for data collecting will be provided to investigators in order to collect data during study visit. This must be attached to patient´s medical record.

6.1.1 Study Flow Chart and Plan

Site staff will retrospectively collect the necessary information from the patient’s medical record to determine eligibility. Patients will be identified consecutively, if eligible, consented and enrolled onto the study.

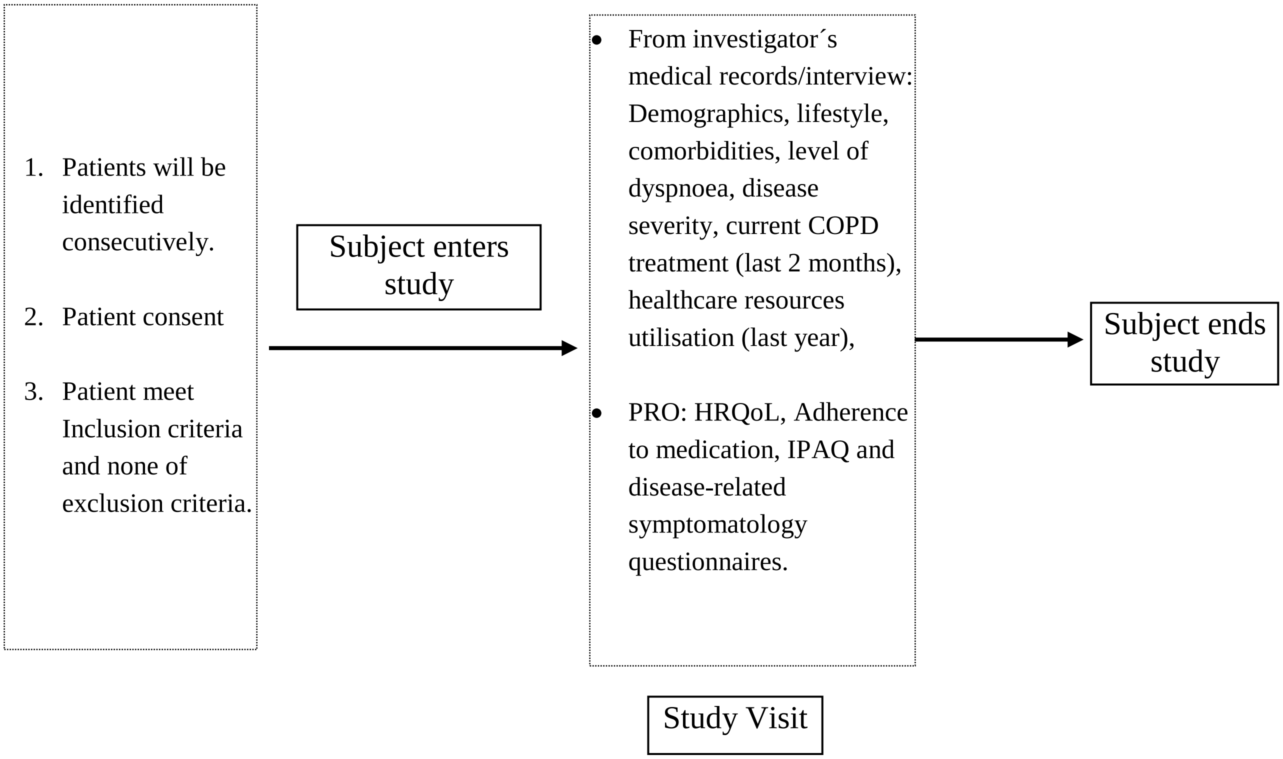

There will be only one study visit at site. The assessments to be conducted in this study are shown in following table.

**Variable**	**Study Visit** Day 1 Clinics	**Questionnaire / Source**	**Information provider**
Informed consent	X	Country ICF	Investigator
Inclusion/exclusion criteria	X	Medical records/interview	Investigator
Socio-demographics, anthropometrics, and socio-economics and lifestyle data	X	Medical records/interview	Investigator
Medical history (COPD, tobacco and comorbidities-COTE)	X	Medical records/interview	Investigator
Medication used for COPD and others last 2 months	X	Medical records/interview	Investigator
COPD exacerbations history in last 12 months (number and severity)	X	Medical records/interview	Investigator
Health resources utilization in the last 12 months	X	Medical records/interview	Investigator

Level of dyspnea (modified MRC dyspnea scale)	X	Medical records/interview	Investigator
Disease classification and severity/prognosis assessment (GOLG and BODEX)		Medical records/interview	Investigator
HRQoL	X	CAT (2009)	PRO
Night-time symptoms	X	NiSCI, 11-item questionnaire version 2014 developed by Evidera Inc	PRO
Day-time symptoms	X	E-RS™, 2009 validated 14-item questionnaire developed by Evidera Inc, (Copyright, All rights reserved 2013).	PRO
Early morning symptoms	X	EMSCI 10-item questionnaire version 2014 developed by Evidera Inc, in validation process by AstraZeneca.	PRO
Adherence to inhalers (TAI and MMAS-8)	X	TAI (on line version 2016) and MMAS-8 (2008)	PRO
Physical activity	X	IPAQ (2003)	PRO

Abbreviations: COPD = chronic obstructive pulmonary disease; MRC = Medical Research Council; GOLD = Global Initiative for Chronic Obstructive Lung Disease; IPAQ = International Physical Activity Questionnaire; CAT = COPD Assessment Test; HADS = Hospital Anxiety and Depression scale; CASIS = COPD and Asthma Sleep Impact Scale; HRU = Healthcare resources utilisation.

6.1.2 Procedures

As already described in previous sections, patients will be enrolled by their physicians who will collect data at the study visit, from medical records or actively interviewing the patient in order to assess all variables. All data should be stated in medical records. In addition, the patient will be asked to provide data through PRO tools.

Any procedure ordered by the physician during this study will be one that is appropriate to the routine clinical care delivered to the patient at the discretion of the participating physician

6.1.2.1 Specific procedures for Investigators´ assessment tools.

a) Disease group (2013 GOLD Recommendation).

Current GOLD report (1) recommend a classification of disease into four Groups (A to D) according symptom´s impact (using specific cutpoints for CAT, or mMRC) and disease´s risk (using specific spirometric or previous exacerbations cutpoints).

GOLD Group definition from 2013 classifies patients in (Appendix 3):

Patient Group A = Low risk, low symptom burden

- Low symptom burden (mMRC of 0-1 or CAT score < 10) AND
- GOLD 1-2 airflow limitation (mild or moderate: FEV_1 of 50% or greater) AND low exacerbation rate (0-1/year)

Patient Group B = Low risk, higher symptom burden

- Higher symptom burden (mMRC of 2 or more or CAT of 10 or more) AND
- GOLD 1-2 airflow limitation (mild or moderate: FEV_1 of 50% or greater) AND low exacerbation rate (0-1/year)

Patient Group C = High risk, low symptom burden

- Low symptom burden (mMRC of 0-1 or CAT score < 10) AND
- GOLD 3-4 airflow obstruction (severe or very severe: FEV_1 < 50%) AND/OR high exacerbation rate (2 or more/year)

D = High risk, higher symptom burden

- Higher symptom burden (mMRC of 2 or more or CAT of 10 or more) AND
- GOLD 3-4 airflow obstruction (severe or very severe: FEV_1 < 50%) AND/OR high exacerbation rate (2 or more/year)

According to clinical practice, bronchodilator test will consist of one Pulmonary Function Test (one set of Forced manoeuvre only for FEV_1 and FVC measurement), followed by the inhalation on 400 mcg of salbutamol through a Spacer device, and followed 10-15 minutes later by another Pulmonary Function Test (one set of Forced manoeuvre only).

Airflow obstruction severity is classified according spirometric classification of COPD based on post-bronchodilator FEV_1 according to the criteria in the following table:

**GOLD Airflow Obstruction**	**Post-Bronchodilator FEV_1**
GOLD I: Mild	$FEV_1/FVC < 0.70$<br>$FEV_1 \geq 80\%$ predicted
GOLD II: Moderate	$FEV_1/FVC < 0.70$<br>$50\% \leq FEV_1 < 80\%$ predicted
GOLD III: Severe	$FEV_1/FVC < 0.70$<br>$30\% \leq FEV_1 < 50\%$ predicted
GOLD IV: Very Severe	$FEV_1/FVC < 0.70$<br>$FEV_1 < 30\%$ predicted

FEV_1: forced expiratory volume in one second; FVC: forced vital capacity;

Spirometry data from the previous 12 months will be valid.

b) Level of dyspnea (modified MRC -mMRC- dyspnea scale)

At study visit, the physician will ask the patient about their perceived breathlessness and will use the MMRC dyspnea scale (26) to classify it into MMRC dyspnea grades (Appendix 4):

Dyspnea Grade:

0 Breathless with strenuous exercise

1 Short of breath when hurrying on the level or walking up a slight hill

2 Walks slower than people of the same age on the level because of breathlessness or stops for breath when walking at own pace on the level

3 Stops for breath after walking about 100 meters or after a few minutes on the level

4 Too breathless to leave the house or breathless when dressing or undressing

NB: This is the modified MRC scale that uses the same descriptors as the original MRC scale in which the descriptors are numbered 1-5.

c) Comorbidities, COTE Index

At study visit, the physician will ask the patient about comorbidities considering those detailed in COTE Index (22). In that index a scale value points in the range of one to six

points was assigned to each selected comorbidity in proportion to its HR (1–1.5 = 1, .1.5–2 = 2,and .2 = 6 points with the exception other cancers, which were assigned two points).

Comorbidities and value points are the following:

- Lung, esophageal, pancreatic, and breast* cancer: 6 points
- Anxiety*: 6 points
- All other cancers: 2 points
- Liver cirrhosis: 2 points
- Atrial fibrillation/flutter: 2 points
- Diabetes with neuropathy: 2 points
- Pulmonary fibrosis: 2 points
- Congestive heart failure: 1 point
- Gastric/duodenal ulcers: 1 point
- Coronary artery disease: 1 point

* Valid on the female population only.

d) BODEx Index

The BODEx index (29) have demonstrated excellent prognostic value. It considers body mass index, airflow obstruction, dyspnea, and frequency of exacerbations. All severe exacerbation episodes requiring hospital management during the previous year to inclusion should be collected.

Severe exacerbation is defined as any sustained increase in respiratory symptomatology vs the patient baseline situation, requiring modification of habitual medication and hospital care (emergency visit or admission).

Patients are divided into three groups according to the recorded frequency of severe exacerbation:

- Group A (patients with no severe exacerbation);
- Group B (patients with one or two severe exacerbation);
- Group C (patients with 3 or more severe exacerbation).

Variable points on BODEx Index

1. FEV1 (% of predicted)
    - ≥65: 0 point
    - 50–64: 1 point
    - 36–49: 2 points
    - ≤35: 3 points

2. mMRC dyspnea scale:

- 0–1: 0 point
- 2: 1 point
- 3: 2 point
- 4: 3 point

3. Body-mass index (BMI, measured as weight^2/height):
   - >21: 0 point
   - ≤21: 1 point

4. Severe exacerbations:
   - Group A (patients with no severe exacerbation): 0 point
   - Group B (patients with one or two severe exacerbation): 1 point
   - Group C (patients with 3 or more severe exacerbation): 2 points

The score range for this index is therefore between 0 and 9 points. The lesser score the better survival.

e) COPD exacerbations history

For this study purposes, a COPD exacerbation is defined as an acute event characterized by a worsening of the patient’s respiratory symptoms that is beyond day-to-day variations and leads to a change in medication. The following severity categories will be used:

Severity	Definition
Mild	Increase of COPD symptoms associated with increased use of bronchodilators
Moderate	Increase of COPD symptoms treated with antibiotics and/or systemic corticosteroids
Severe	Increase in COPD symptoms which leads to hospitalization (overnight stay at hospital or emergency room)

At study visit, information to be recorded regarding COPD exacerbation during previous year includes number, need of ambulatory treatment with or without antibiotics and/or systemic corticosteroids, emergency room treatment or hospitalization.

6.1.2.2 Specific procedures for Patient reported Outcomes questionnaires.

Some variables will be collected directly from patients through PRO questionnaires. The patients will complete the questionnaires in a printed paper version. The patient should be informed about the purpose and importance of completing the questionnaires and be given adequate time to complete all items. The questions should be completed in a quiet place without influence from family, friends, or study personnel, in a pre-defined order

a) Day-time, Morning and Night-time Symptoms of COPD questionnaire. (Appendix 2).

"Evaluating Respiratory Symptoms in COPD" E-RS™ 2016 (formerly known as EXACT-RS) is a Day-time Symptoms validated questionnaire of 14 items developed by Evidera Inc to which AstraZeneca has approved and payed access. This tool asks about COPD symptoms during day time. For this study purpose, as this is cross sectional one visit study, this questionnaire is going to assess symptoms occurred from starting regular daytime activities to the moment that patient went to sleep of previous day of study visit day.

The Night-time, and Early Morning Symptoms of COPD will be assessed through Nighttime Symptoms of COPD Instrument (NiSCI) and Early Morning Symptoms of COPD Instrument (EMSCI). Both developed by Evidera Inc currently in validation process by AstraZeneca, 11 and 10 items questionnaire respectively.

NiSCI assess symptoms occurred during time from when patients went to bed previous night until they woke up and go out of bed to start their daily living activities of the day of study visit.

EMSCI assess symptoms occurred during time from when patients got out of bed to start their daily living activities to the moment they are ready for regular daytime activities of the day of study visit.

b) Adherence to respiratory medicines, TAI and MMAS-8 Questionnaires. (Appendix 5)

TAI Questionnaires (14) includes 12 items. The first patient´s self-administered 10 items are scored from 1 to 5 (where 1 = worst possible score and 5 = best possible score). Total score ranges between 10 and 50 for the 10-items TAI, and between 12 and 54 for the 12-items TAI.

Adherence is rated as:

- Good adherence: 50 points
- Intermediate adherence: 46 to 49 points
- Poor adherence: < 45 points

The final two items are completed by the healthcare professionals and scored as 1 or 2 (where 1 = bad and 2 = good). These items were designed to identify unwitting non-adherent behavior (failure in understanding medication use, dosage or inhalation technique).

The eight-item Morisky Medication Adherence Scale (MMAS-8) is a structured self-report (33). The Morisky scale is composed of 8 questions related to the use of medication as prescribed by a patient's physician: 7 .Yes (score, 0) and No (score, 1) questions and 1 question with 5 possible answers Likert scale type (0-4).

Total score ranges from 0 to 8. Answering no indicates better adherence. For question 8 result (0-4 points) should be divided by 4 to standardize punctuation. Patient adherence is classified into 3 categories based on responses to the MMAS scale:

Adherence is rate as:

- High adherence (score, 8)
- Medium adherence (score, 7-6)
- Low adherence (score, < 6)

c) COPD Assessment Test (CAT). (Appendix 6)

The CAT is a short validated patient-completed questionnaire (31) that has been designed to provide a simple and reliable measure of health status in COPD (Appendix 6).

The CAT questionnaire consists of eight items, each formatted as a semantic six-point differential scale, making the tool easy to administered and easy for patients to complete. The eight items cover a wide range of COPD severity and can be scored as a single scale, with scores ranging from 0 to 40; where higher scores represent worse health status. The content covers: cough, phlegm, chest tightness, breathlessness going up hills/stairs, activity limitation at home, confidence leaving home, sleep and energy.

d) International Physical Activity Questionnaire (IPAQ). (Appendix 7)

IPAQ (32) assesses physical activity undertaken across a comprehensive set of domains including:

- leisure time physical activity
- domestic and gardening (yard) activities
- work-related physical activity
- transport-related physical activity;

The IPAQ will be used in the short form (4 generic items) because it is more appropriate in an observational study.

The IPAQ short form asks about three specific types of activity undertaken in the four domains introduced above. The specific types of activity that are assessed are walking, moderate-intensity activities and vigorous-intensity activities. It includes a sitting question which is an additional indicator variable of time spent in sedentary activity.

The items in the short IPAQ form are structured to provide separate scores on walking, moderate-intensity and vigorous-intensity activity. Computation of the total score for the short form requires summation of the duration (in minutes) and frequency (days) of walking, moderate-intensity and vigorous-intensity activities. Domain specific estimates cannot be

estimated. The IPAQ sitting question is not included as part of any summary score of physical activity (Appendix 7).

6.1.2.3 Direct Medical Healthcare Resources Utilisation (HRU)

Healthcare resources utilization during last year will be collected at study visit (number of visits to primary care or specialists) as stated in medical records. These questions will be specified in the eCRF.

6.1.2.4 Current therapy for COPD and others

The Investigator will enter the following information into the eCRF, regarding the current COPD medication, taken during past two months:

- Therapeutic class (all inclusive): short-acting β_2-agonists – SABAs; short-acting muscarinic antagonists – SAMAs; long-acting β_2-agonists – LABAs; long-acting muscarinic antagonists – LAMAs; inhaled glucocorticosteroids – ICS; methylxanthines, systemic glucocorticosteroids, phosphodiesterase 4 inhibitors; leukotriene receptor antagonists, mucolytics and Oxygen.
- Drug name of each therapeutic class used
- Modality of use: rescue/maintenance
- Daily doses

6.1.3 Quality Control

Monitoring

Before the first subject is recruited into the study, the local Marketing Company, or CRO Representative will:

- Establish the adequacy of the facilities and the investigator's capability to appropriately select the sample
- Discuss with the investigator(s) (and other personnel involved with the study) their responsibilities with regards to protocol compliance, and the responsibilities of AstraZeneca or its representatives. This will be documented in a NIS Primary Agreement between AstraZeneca/delegate and the investigator.

During the study the local MC representative or delegate can implement different activities to assure compliance with AZ standards of quality. These activities could include but are not limited to:

Contacts with the sites to:

- Provide information and support to the investigator(s)
- Confirm that the research team is complying with the protocol and that data are being accurately recorded in the electronic case report forms (eCRFs)

- Ensure that the subject informed consent forms are signed and stored at the investigator's site

Monitoring activities for:

- Checking a sample of ICFs
- Checking that subjects exist in medical records (a sample)

Because of the non-interventional nature of this study, a simple monitoring plan is required. 100% of source data verification (SDV) of 10% of total enrolled patients, estimating that one site per country would be enough. Local AZ marketing companies will select the highest recruitment potential centre to be monitored. Monitoring visit will be conducted by the CRO immediately after all patients are enrolled in that centre to perform 100% SDV.

Regional and local study team and National Coordinators will monitor electronically through website study platform each site since the first patient inn.

Different signals (e.g., high rejection rate in a site) should be used as potential identification of low protocol compliance by investigators.

If these or any other signal occurs or if the local coordinator is suspicious of a potential non-optimal level of protocol compliance by the site investigator, specific measures should be adopted to evaluate the situation, identify the issue and implement specific action plans to correct the situation.

Representatives of the Sponsor's quality assurance unit/monitoring team and competent regulatory authorities must be permitted to inspect all study-related documents and other materials at the site, including the Investigator Site File, the completed eCRFs and the patient's original medical records. Audits may be conducted at any time during or after the study to ensure the validity and integrity of the study data.

Training of Study Site Personnel

- At least one meeting with CRO and local AZ Medical staff will be held prior to the study start, and periodical meetings will be held through the study with the CRO and AZ LatAm regional coordination

- A coordinator's meeting will be held remotely to review the protocol prior to the study start and will be led by AZ LatAm regional coordination.

- An investigator meeting per country will be held at local AZ offices. Local AZ medical staff will be responsible of inviting and logistics. Training will be conducted by National Coordinators, CRO and AZ study team.

- Initiation visits will be performed during the investigator meeting by CRO and will include detailed review of protocol, eCRF and patients' questionnaires, study procedures and study calendar. For those PI who cannot attend to the investigator meeting, initiation will be performed remotely by phone.

- The activation of centres will be done by mail and personally by phone. This would means the kick off for enrolment. All regulatory and study files should be on place and initiation and investigator meeting performed.

The Principal Investigator will ensure that appropriate training relevant to the NIS is given to investigational staff, and that any new information relevant to the performance of this NIS is forwarded to the staff involved.

6.1.4 Storage and Retention

Upon completion of Data Base Lock and at the agreed time point, data from the LASSYC Study database will be transferred to AZ via a secure file transfer portal in the pre agreed format.

All original source documentation is expected to be stored at the site for the longest possible time as required by local applicable regulations or as specified in the contract, whichever is longer. The records must be available for review in the event the site is selected for monitoring, audits, or inspections and must be safely archived following the study conclusion, according to local regulations or as specified in the contract, whichever is longer.

Essential documents, as listed below, must be retained by the investigator for as long as needed to comply with national regulations. Essential documents include:

- IRB/IEC approvals for the study protocol and all amendments
- All source documents
- eCRF contents
- Patients' or next of kin/legal representative's ICFs (with study number and title)
- Any other pertinent study document.

AZ will notify the investigators/institutions when the study-related records are no longer required. The investigator agrees to adhere to the document retention procedures by signing the protocol. In the event that archiving of the file is no longer possible at the site, the site will be instructed to notify AZ.

6.2 Protection of Human Subjects

The Non-Interventional Study will be performed in accordance with ethical principles that are consistent with the Declaration of Helsinki, ICH GCPs, GPP and the applicable legislation on Non-Interventional Studies.

The Investigator will perform the NIS in accordance with the regulations and guidelines governing medical practice and ethics in the country of the NIS and in accordance with currently acceptable techniques and know-how.

The final protocol of the Non-Interventional Study, including the final version of the Subject Informed Consent Form, must be approved or given a favourable opinion in writing by the Ethics Committee/Institutional Review Board (IRB)/Independent Ethics Committee (IEC).

The Ethics Committee/IRB/IEC must also approve any amendment to the protocol and all advertising used to recruit subjects for the study, according to local regulations.

6.2.1 Patient Informed Consent

The Investigator at each site will ensure that the patient is given full and adequate oral and written information about the nature, purpose, possible risk and benefit of the NIS. Patients must also be notified that they are free to discontinue from the NIS at any time. The patients should be given the opportunity to ask questions and allowed time to consider the information provided.

The signed and dated subject informed consent must be obtained before any specific procedure for the NIS is performed, including:

- Interview with the investigator
- Fulfil the questionnaires
- eCRFs completion.

The Investigator must store the original, signed Subject Informed Consent Form and the copy of the signed Subject Informed Consent Form must be given to the subject.

6.2.2 Confidentiality of Study/Subject Data

The patient Informed Consent Form (ICF) will incorporate wording that complies with relevant data protection and privacy legislation. Pursuant to this wording, subjects will authorise the collection, use and disclosure of their personal data by the Investigator and by those persons who need that information for the purposes of the NIS.

The patient Informed Consent Form will explain that NIS data will be stored in a computer database, maintaining confidentiality in accordance with the local law for Data Protection.

The patient Informed Consent Form will also explain that for quality check purposes, a monitor of AZ or a monitor of company representing AZ, will require direct access to the signed subject informed consent forms. In case source data verification will be planned as quality check, the Subject Informed Consent Form will explain that for data verification purposes, monitor of AZ or a monitor of company representing AZ may require direct access to source documents that are part of the hospital or practice records relevant to the Non-Interventional Study.

Appendix 1 details template text for this NIS´ICF.

6.3 Management and Report of Adverse Events/Adverse Drug Reactions

6.3.1 Definition of Adverse Events (AE)

An adverse event is the development of an undesirable medical condition or the deterioration of a pre-existing medical condition following or during exposure to a pharmaceutical product, whether or not considered causally related to the product..

The term AE is used to include both serious and non-serious AEs.

6.3.2 Definition of Serious Adverse Events (SAE)

A serious adverse event is an AE occurring during any study phase (i.e., run-in, treatment, washout, follow-up), that fulfils one or more of the following criteria:

- Results in death
- Is immediately life-threatening
- Requires in-patient hospitalisation or prolongation of existing hospitalisation
- Results in persistent or significant disability/incapacity or substantial disruption of the ability to conduct normal life functions
- Is a congenital abnormality or birth defect
- Is an important medical event that may jeopardise the subject or may require medical intervention to prevent one of the outcomes listed above.

6.3.3 Collection of Adverse Events

Not applicable. LASSYC Study is an observational study. AstraZeneca will not supply any products in research of this study.

6.3.4 Reporting of Adverse Events

The LASSYC study is a disease registry. Although information about past and current drug history will be collected, the focus of the study is not on the medicinal products. Moreover, the current study is observational and therefore the clinical practice and patient pathways should be as close as possible with routine practice. All investigators are encouraged to report any observed ADR or serious ADR according to local regulatory requirements and, if the investigator considers it appropriate, to AZ (in case of ADRs of an AZ-product) or the corresponding marketing authorization holder of the drug.

6.4 Study Governance and Committees

A governance structure will be set up including internal and external qualified individuals with relevant experience and expertise for this NIS.

- Internal project members:
    - **AZ LatAm Regional Project** Team with overall scientific responsibility and executive roles responsible for delivering the study.

- **AZ Local Study Teams** integrated by therapeutic medical delegates and other locally designated members of cross functional team, responsible of local operational procedures.

- External project members:
  - A **Scientific Steering Committee** will be set up to support and oversee the study. Constituted by external experts pulmonologists with robust expertise in COPD field. The general role of its members will be to provide scientific support for the study at the following levels:

    - Design and development of the main study documents (study protocol, ICF template and eCRF).
    - Design and development of the Statistical Analysis plan
    - Running of this study in his/her country and at region level (by giving support to regional or local coordination).
    - Interpretation of the results of the study.
    - Design of the communication and publication plan of the study
    - Active participation of publications at regional level and presentation of results at scientific meetings or regional and international congresses.

    The members of LASSYC study's Steering Committee are:

    - Chairman: Dr. Marc Miravitlles (Spain)
    - Co Chairman: Dr. Montes de Oca, María (Venezuela)

    Committee Members:
    - Dra. Ana Menezes (Brazil)
    - Dra. Lopez Varela, Maria Victorina (Uruguay)
    - Dr. Alejandro Casas (Colombia)
    - Dra. Alejandra Ramirez Venegas (México)
    - Dra. Laura Mendoza (Chile)
    - Dra. Ana María Lopez (Argentina)
    - Dr. Luis Ugalde (Costa Rica)

  - **National Study Coordinators:** In those countries where the study will be implemented members of Steering Committee will also assume the role of National Coordinator in order to provide scientific local support and guarantee of protocol compliance throughout the conducting phase.

    National Coordinators will assume the role of national principal investigator, although because of the aforementioned purpose they cannot be contracted as local investigator for patient recruitment in this same study.

    National Coordinators will closely support the Local Study Team.

Specific responsibilities are:

- Guidance on investigator/sites selection in local country
- Led local investigator meeting (one per country) with active participation in the investigator´s training
- Support to the recruitment follow up
- Promotion of timelines compliance
- Scientific support to any concern or query raised locally around protocol and study conduction.

A Principal Investigator will be nominated at each investigational site. The names, centres and *curricula vitae* of all Investigators participating in the study will be detailed in the Clinical Study Report.

6.5 Communication Plan

6.5.1 Publication Plan

In alignment with AZ policies, AZ will prepare a Study Report within 12 months after completion of the LPI (study completion). All reporting will be consistent with the STrengthening the Reporting of OBservational studies in Epidemiology (STROBE) Initiative checklist for cohort studies (STROBE 2012). Reports will be prepared for each of the major analysis steps specified in the protocol. The final report will encompass all planned analyses, including a description of the complete study population, as described above and in the corresponding SAPs.

Final results of the core study will be disseminated through submission of manuscripts for publication and guided by the Uniform Requirements for Manuscripts Submitted to Biomedical Journals: Writing and Editing for Biomedical Publication of the International Committee of Medical Journal Editors (ICMJE).

Publication of data subsets from individual institutions participating in multicentre studies should not precede the primary manuscript(s) on the same topic(s). Selected interim and final results may also be disseminated through publication or presentation at scientific meetings with support as relevant from the study Scientific and Executive Committees.

The sponsor aims to publish at least the main study results in a full text paper in an international peer review journal, after having available the study final report reviewed and signed by the study coordinator and through a manuscript authored by the study Steering Committee. This publication is intended to be submitted in the six months following the issue of the final report.

Abstracts to international congresses containing study result highlights previous to the full text publication could come if the congress calendar offers adequate submission timeframes and with previous approval of Steering Committee.

6.5.2 Compliance with Study Registration and Results Posting Requirements

AZ is committed to providing full and transparent disclosure of, and open access to, the findings of all AZ sponsored studies and information on ongoing studies sponsored by AstraZeneca.

AZ or the delegated CRO must register all qualifying studies prior to enrolment of the first patient, referred to as the First Patient In date. Studies are registered on ClinicalTrials.gov (sponsored by the National Institute of Health) and other country-specific or regional websites as required by law, with study information set forth on AZ internal templates. In addition to publicly registering studies on ClinicalTrials.gov and other country specific or regional websites, basic study information is also posted on AstraZenecaClinicalTrials.com.

Once a study is initially registered, any changes related to study status or protocol amendments must be updated to ensure accurate reporting of all required information. By law (FDA Amendment Act 2007), any changes in a study´s overall recruitment status must be updated on ClinicalTrials.gov no later than 30 days after the change in status. All other changes to posted information must be updated at least quarterly.

Results are disclosed on ClinicalTrials.gov, AstraZenecaClinicalTrials.com and on other public websites in a format and to timelines as required by law and should be submitted by AZ as the Sponsor

6.5.3 Compliance with Financial Disclosure Requirements

Financial compensation will be provided to cover all study procedures under the responsibility of the Investigator. This compensation rate will be determined according to recommended fair market value for the corresponding study activities. Financial disclosure of this compensation will fulfil applicable local laws, codes and regulations.

Once CROs´ contract is in place, study will begin start up process according services, timelines and financial aspects detailed in Project Agreement of MSA.

Local AstraZeneca marketing companies shall set up local contracts with sites´ investigators and local national coordinators detailing financial aspects, services and timelines for the local study implementation.

6.5.4 Changes to Protocol

Changes to the protocol will be documented in written protocol amendments. Major (i.e. substantial, significant) amendments will be approved by the relevant regulatory authorities and will usually require submission or notification to the relevant IRB/IEC for approval or favorable opinion, if applicable. In such cases, the amendment will be implemented at the site only after approval or favorable opinion has been obtained.

Minor (non-substantial) protocol amendments, including administrative changes, will be filed at each participating site and will be submitted to the relevant IRB/IEC or regulatory

authorities where required by pertinent regulations. Any amendment that could have an impact on the patient's agreement to participate in the study requires the patient's informed consent prior to continued participation in the study.

Amendments and updates to the protocol will be documented in Section .Amendment History.

7. APPENDICES

7.1 APPENDIX 1: PATIENT INFORMATION SHEET AND INFORMED CONSENT FORM (ICF)

The following is a template to be used as model to redact ICF locally according to local regulations and laws.

Título: Estudio Latinoamericano de los Síntomas durante 24 hs en pacientes con Enfermedad Pulmonar Obstructiva Crónica

Código del estudio: D2287R0012

Patrocinador del Estudio: AstraZeneca

Estimado paciente:

Ha sido invitado a participar en un estudio sobre la *enfermedad pulmonar obstructiva crónica (EPOC)*. Este documento se denomina consentimiento informado. Contiene una explicación completa del estudio en el que se le invita a participar y debería leerlo atentamente y comentar con el médico cualquier aspecto que no comprenda. Podrá hacer al médico del estudio todas las preguntas que quiera. Si decide participar en el estudio, se le pedirá que firme y feche el documento de consentimiento.

Se le pide que participe en este estudio, que está patrocinado por AstraZeneca, porque ha sido diagnosticado de EPOC y porque su médico está participando también en el estudio. En este estudio participarán alrededor de 900 pacientes.

NATURALEZA Y OBJETIVO DEL ESTUDIO

AstraZeneca lleva a cabo este estudio para saber más sobre la EPOC y los síntomas asociados en siete países de Latino America.

Este estudio NO implica tomar un medicamento nuevo o en investigación. Si durante la visita de hoy su médico considera que le convendría tomar algún medicamento en particular que fuera adecuado para usted, se lo recetará para su enfermedad como parte de la práctica médica habitual y no por participar en este estudio de seguimiento.

PROCEDIMIENTOS DEL ESTUDIO

Si decide participar en este estudio, se le pedirá que complete 7 cuestionarios sobre los

siguientes aspectos: intensidad de los síntomas (nocturnos, diurnos y a primera hora de la mañana), calidad de vida, actividad física, y uso de sus medicamentos respiratorios. Su médico también registrará algunos datos sobre su estado y anotará los tipos de medicamentos que está tomando para su EPOC.

Los datos del estudio se recopilarán durante la entrevista personal (en el momento de su inclusión en el estudio) con su médico.

Si decide participar en este estudio, tiene que saber que se utilizarán algunos datos relacionados con su salud. Sus documentos médicos podrían ser revisados por personas dependientes de las autoridades sanitarias, miembros de los comités éticos independientes y otras personas designadas por ley para comprobar que el estudio se está llevando a cabo correctamente.

RIESGOS Y MOLESTIAS

Participar en este estudio no entraña ningún riesgo para usted, ya que no se llevarán a cabo pruebas médicas adicionales ni se administrarán otros tratamientos distintos de los que formen parte de la práctica clínica normal.

POSIBLES EFECTOS BENEFICIOSOS DEL ESTUDIO

Este estudio se hace únicamente con fines de investigación. La información que se obtenga en este estudio puede ayudar a otras personas en el futuro.

COMPENSACIÓN

No recibirá remuneración alguna por participar en este estudio y no obtendrá ningún beneficio directo de su participación en él.

El médico a cargo del estudio será compensado por AstraZeneca por su labor para recopilar datos.

PARTICIPACIÓN VOLUNTARIA / RETIRADA DEL ESTUDIO

Su participación es totalmente voluntaria. Es usted libre de negarse a participar en este estudio sin que eso afecte a su asistencia futura.

POLÍTICA DE CONFIDENCIALIDAD

Los datos obtenidos sobre usted mientras participe en este estudio, así como los documentos sanitarios relacionados, se mantendrán en la más estricta confidencialidad en todo momento. No obstante, tendrán que facilitarse a terceros que trabajen por cuenta de AstraZeneca, a los miembros del Comité Ético de Investigación Clínica y a las autoridades encargadas de regular los medicamentos.

Al firmar el impreso de consentimiento, autoriza este acceso durante el estudio actual y

cualquier investigación ulterior que pueda realizarse. No obstante, AstraZeneca adoptará medidas para proteger su información personal y no incluirá su nombre en impresiones del patrocinador, informes ni publicaciones, ni lo divulgará en el futuro. Durante el estudio, tiene la garantía de que se cumplirá con todas las leyes locales. Si se retira del estudio, no podremos recoger su información personal, pero es posible que tengamos que continuar utilizando la información que ya hayamos recopilado.

Los datos se enviarán a otros países, pero no se mencionará su nombre ni se le identificará en ningún informe o publicación, ni se presentarán datos que permitan identificarle. No obstante, AstraZeneca mantiene un alto grado de confidencialidad y de protección de la información.

Se obtendrán y procesarán datos personales, sólo con fines de investigación. AstraZeneca (que controlará el uso de los datos) adoptará medidas para garantizar la protección de sus datos personales. Al participar en este estudio, accede a no restringir el uso de ningún dato, aun cuando se retire del estudio, y autoriza la transferencia de sus datos personales a AstraZeneca y a las autoridades encargadas de la regulación de los medicamentos, tanto dentro como fuera de su país.

LOS DERECHOS QUE LE RECONOZCA CUALQUIER LEGISLACIÓN SOBRE PROTECCIÓN DE DATOS VIGENTE NO RESULTARÁN AFECTADOS

FUENTES DE INFORMACIÓN ADICIONAL

Si tiene alguna pregunta o alguna duda sobre cualquier aspecto del estudio, o si necesita ayuda para un problema de salud relacionado con el estudio, no dude en contactar con su médico a cargo del estudio:

Dr. .... Teléfono: ....

Yo,...

(Nombre completo del paciente)

He leído la hoja informativa que se me ha entregado

He podido preguntar acerca del estudio.

He recibido información suficiente sobre el estudio.

He hablado con ....

(Nombre completo del investigador)

7.2 APPENDIX 2: NIGHT-TIME, MORNING AND DAY-TIME SYMPTOMS OF COPD

7.2.1 "Evaluating Respiratory Symptoms in COPD" (E-RS™) Questionnaire. To be used for daytime symptoms of day before to study visit

Description	Required Text	Translation
Title	E-RS	E-RS
DD	Daily Diary	Daily Diary
Q 1 of 14	Question 1 {1} of 14	Question 1 {1} of 14
Instructions	As you answer the following questions, please select the option that best describes your experience.	As you answer the following questions, please select the option that best describes your experience.
1	Did your chest feel congested today?	Did your chest feel congested today?
	Not at all	Not at all
	Slightly	Slightly
	Moderately	Moderately
	Severely	Severely
	Extremely	Extremely
2	How often did you cough today?	How often did you cough today?
	Not at all	Not at all
	Rarely	Rarely
	Occasionally	Occasionally
	Frequently	Frequently
	Almost constantly	Almost constantly
3	How much mucus (phlegm) did you bring up when coughing today?	How much mucus (phlegm) did you bring up when coughing today?

Description	Required Text	Translation
	None at all	None at all
	A little	A little
	Some	Some
	A great deal	A great deal
	A very great deal	A very great deal
4	How difficult was it to bring up mucus (phlegm) today?	How difficult was it to bring up mucus (phlegm) today?
	Not at all	Not at all
	Slightly	Slightly
	Moderately	Moderately
	Quite a bit	Quite a bit
	Extremely	Extremely
5	Did you have chest discomfort today?	Did you have chest discomfort today?
	Not at all	Not at all
	Slight	Slight
	Moderate	Moderate
	Severe	Severe
	Extreme	Extreme
6	Did your chest feel tight today?	Did your chest feel tight today?
	Not at all	Not at all
	Slightly	Slightly
	Moderately	Moderately
	Severely	Severely

Description	Required Text	Translation
	Extremely	Extremely
7	Were you breathless today?	Were you breathless today?
	Not at all	Not at all
	Slightly	Slightly
	Moderately	Moderately
	Severely	Severely
	Extremely	Extremely
8	Describe how breathless you were today:	Describe how breathless you were today:
	Unaware of breathlessness	Unaware of breathlessness
	Breathless during strenuous activity	Breathless during strenuous activity
	Breathless during light activity	Breathless during light activity
	Breathless when washing or dressing	Breathless when washing or dressing
	Present when resting	Present when resting
9	Were you short of breath today when performing your usual personal care activities like washing or dressing?	Were you short of breath today when performing your usual personal care activities like washing or dressing?
	Not at all	Not at all
	Slightly	Slightly
	Moderately	Moderately
	Severely	Severely
	Extremely	Extremely
	Too breathless to do these	Too breathless to do these
10	Were you short of breath today when performing your usual indoor activities	Were you short of breath today when performing your usual indoor

Description	Required Text	Translation
	like cleaning or household work?	activities like cleaning or household work?
	Not at all	Not at all
	Slightly	Slightly
	Moderately	Moderately
	Severely	Severely
	Extremely	Extremely
	Too breathless to do these	Too breathless to do these
11	Were you short of breath today when performing your usual activities outside the home such as yard work or errands?	Were you short of breath today when performing your usual activities outside the home such as yard work or errands?
	Not at all	Not at all
	Slightly	Slightly
	Moderately	Moderately
	Severely	Severely
	Extremely	Extremely
	Too breathless to do these	Too breathless to do these
12	Were you tired or weak today?	Were you tired or weak today?
	Not at all	Not at all
	Slightly	Slightly
	Moderately	Moderately
	Severely	Severely
	Extremely	Extremely

Description	Required Text	Translation
13	Last night, was your sleep disturbed?	Last night, was your sleep disturbed?
	Not at all	Not at all
	Slightly	Slightly
	Moderately	Moderately
	Severely	Severely
	Extremely	Extremely
14	How scared or worried were you about your lung problems today?	How scared or worried were you about your lung problems today?
	Not at all	Not at all
	Slightly	Slightly
	Moderately	Moderately
	Severely	Severely
	Extremely	Extremely
Copyright	E-RS™ 2016, Evidera, Inc. All rights reserved.	E-RS™ 2016, Evidera, Inc. All rights reserved.

7.2.2 NiSCI Questionnaire.

To be used for night time symptoms of time from when patients went to bed previous night until they woke up and go out of bed to start their daily living activities of the day of study visit.

---

1. ¿Se despertó anoche debido a los síntomas de la EPOC?

- ☐ No
- ☐ Sí

1a. ¿Cuántas veces se despertó debido a los síntomas de la EPOC?

_____ veces

---

2. ¿Tuvo anoche alguno de los siguientes?

**2a. Tos**	☐ No	☐ Sí
**2b. Silbidos en el pecho**	☐ No	☐ Sí
**2c. Falta de aire**	☐ No	☐ Sí
**2d. Opresión en el pecho**	☐ No	☐ Sí
**2e. Congestión en el pecho**	☐ No	☐ Sí
**2f. Dificultad para expulsar las flemas**	☐ No	☐ Sí

Usted indicó que anoche tuvo tos...

2a.i. ¿Qué intensidad tuvo la tos?

- ☐ Leve
- ☐ Moderada
- ☐ Intensa
- ☐ Muy intensa

Usted indicó que anoche tuvo silbidos en el pecho...

2b.i. ¿Qué intensidad tuvieron los silbidos en el pecho?

- ☐ Leve
- ☐ Moderada
- ☐ Intensa
- ☐ Muy intensa

Usted indicó que anoche tuvo falta de aire...

2c.i. ¿Qué intensidad tuvo la falta de aire?

- ☐ Leve
- ☐ Moderada
- ☐ Intensa
- ☐ Muy intensa

Usted indicó que anoche tuvo opresión en el pecho...

2d.i. ¿Qué intensidad tuvo la opresión en el pecho?

- ☐ Leve
- ☐ Moderada
- ☐ Intensa
- ☐ Muy intensa

Usted indicó que anoche tuvo congestión en el pecho...

2e.i. ¿Qué intensidad tuvo la congestión en el pecho?

- ☐ Leve
- ☐ Moderada
- ☐ Intensa
- ☐ Muy intensa

Usted indicó que anoche tuvo dificultad para expulsar las flemas...

2f.i. ¿Qué intensidad tuvo la dificultad para expulsar las flemas?

- ☐ Leve
- ☐ Moderada
- ☐ Intensa
- ☐ Muy intensa

---

3. En general, ¿qué intensidad tuvieron los síntomas de la EPOC anoche?

- ☐ No tuve ningún síntoma

- ☐ Leve
- ☐ Moderada
- ☐ Intensa
- ☐ Muy intensa

---

4. ¿Cuántos disparos se administró anoche de su medicamento de alivio rápido?

_____ disparos

7.2.3 EMSCI Questionnaire.

To be used for early morning symptoms from when patients got out of bed to start their daily living activities to the moment they are ready for regular daytime activities of the day of study visit.

1. ¿Tuvo esta mañana alguno de los siguientes?

**1a. Tos**	☐ No	☐ Sí
**1b. Silbidos en el pecho**	☐ No	☐ Sí
**1c. Falta de aire**	☐ No	☐ Sí
**1d. Opresión en el pecho**	☐ No	☐ Sí
**1e. Congestión en el pecho**	☐ No	☐ Sí
**1f. Dificultad para expulsar las flemas**	☐ No	☐ Sí

Usted indicó que esta mañana tuvo tos...

1a.i. ¿Qué intensidad tuvo la tos?

- ☐ Leve
- ☐ Moderada
- ☐ Intensa

- ☐ Muy intense

Usted indicó que esta mañana tuvo silbidos en el pecho...

1b.i. ¿Qué intensidad tuvieron los silbidos en el pecho?

- ☐ Leve
- ☐ Moderada
- ☐ Intensa
- ☐ Muy intensa

Usted indicó que esta mañana tuvo falta de aire...

1c.i. ¿Qué intensidad tuvo la falta de aire?

- ☐ Leve
- ☐ Moderada
- ☐ Intensa
- ☐ Muy intensa

Usted indicó que esta mañana tuvo opresión en el pecho...

1d.i. ¿Qué intensidad tuvo la opresión en el pecho?

- ☐ Leve
- ☐ Moderada
- ☐ Intensa
- ☐ Muy intensa

Usted indicó que esta mañana tuvo congestión en el pecho...

1e.i. ¿Qué intensidad tuvo la congestión en el pecho?

- ☐ Leve
- ☐ Moderada
- ☐ Intensa

- ☐ Muy intensa

Usted indicó que esta mañana tuvo dificultad para expulsar las flemas...

1f.i. ¿Qué intensidad tuvo la dificultad para expulsar las flemas?

- ☐ Leve
- ☐ Moderada
- ☐ Intensa
- ☐ Muy intensa

---

2. En general, ¿qué intensidad tuvieron los síntomas de la EPOC esta mañana?

- ☐ No tuve ningún síntoma
- ☐ Leve
- ☐ Moderada
- ☐ Intensa
- ☐ Muy intensa

---

3. ¿Hasta qué punto ha limitado sus actividades esta mañana debido a los síntomas de la EPOC?

- ☐ Nada
- ☐ Un poco
- ☐ Moderadamente
- ☐ Mucho
- ☐ Muchísimo

---

4. ¿Cuántos disparos se administró esta mañana de su medicamento de alivio rápido?

_____ disparos

Attachments

7.3 APPENDIX 3: DISEASE SEVERITY (2013 GOLD DOCUMENT)

(To be used only by the investigator)

GOLD Group definition from 2013 classifies patients in:

Patient Group A = Low risk, low symptom burden

- Low symptom burden (mMRC of 0-1 or CAT score < 10) AND
- GOLD 1-2 airflow limitation (mild or moderate: FEV_1 of 50% or greater) AND low exacerbation rate (0-1/year)

Patient Group B = Low risk, higher symptom burden

- Higher symptom burden (mMRC of 2 or more or CAT of 10 or more) AND
- GOLD 1-2 airflow limitation (mild or moderate: FEV_1 of 50% or greater) AND low exacerbation rate (0-1/year)

Patient Group C = High risk, low symptom burden

- Low symptom burden (mMRC of 0-1 or CAT score < 10) AND
- GOLD 3-4 airflow obstruction (severe or very severe: FEV_1 < 50%) AND/OR high exacerbation rate (2 or more/year)

D = High risk, higher symptom burden

- Higher symptom burden (mMRC of 2 or more or CAT of 10 or more) AND
- GOLD 3-4 airflow obstruction (severe or very severe: FEV_1 < 50%) AND/OR high exacerbation rate (2 or more/year).

According to clinical practice, bronchodilator test will consist of one Pulmonary Function Test (one set of forced manoeuvres only for FEV_1 and FVC measurement), followed by the inhalation on 400 mcg of salbutamol through a Spacer device, and followed 10-15 minutes later by another Pulmonary Function Test (one set of forced manoeuvres only).

Airflow obstruction severity is classified according spirometric classification of COPD based on post-bronchodilator FEV_1 according to the criteria in the following table.

**GOLD Airflow Obstruction**	**Post-Bronchodilator FEV_1**
GOLD I: Mild	$FEV_1/FVC < 0.70$<br>$FEV_1 \geq 80\%$ predicted
GOLD II: Moderate	$FEV_1/FVC < 0.70$<br>$50\% \leq FEV_1 < 80\%$ predicted
GOLD III: Severe	$FEV_1/FVC < 0.70$<br>$30\% \leq FEV_1 < 50\%$ predicted
GOLD IV: Very Severe	$FEV_1/FVC < 0.70$<br>$FEV_1 < 30\%$ predicted

FEV_1: forced expiratory volume in one second; FVC: forced vital capacity.

7.4 APPENDIX 4: MODIFIED MRC DYSPNEA SCALE, mMRC

(To be used only by the investigator).

Grade mMRC

0 Breathless with strenuous exercise.

1 Short of breath when hurrying on the level or walking up a slight hill.

2 Walks slower than people of the same age on the level because of breathlessness or stops for breath when walking at own pace on the level.

3 Stops for breath after walking about 100 meters or after a few minutes on the level.

4 Too breathless to leave the house or breathless when dressing or undressing.

7.5 APPENDIX 5: ADHERENCE QUESTIONNAIRES

7.5.1 TAI Questionnaire:

Includes 12 items. The first patient´s self-administered 10 items and 2 last are answered by investigator.

Patient domain: questions, responses (scores)

1. During the last 7 days, how many times did you forget to take your usual inhalers?
   All (1) More than half (2) Approximately a half (3) Less than half (4) None (5)

2. Do you forget to take inhalers?
   Always (1) Mostly (2) Sometimes (3) Rarely (4) Never (5)

3. When you feel good about your illness, do you stop taking your inhalers?
   Always (1) Mostly (2) Sometimes (3) Rarely (4) Never (5)

4. When you are on vacation or weekend, do you stop taking your inhalers?
   Always (1) Mostly (2) Sometimes (3) Rarely (4) Never (5)

5. When you are nervous or sad, do you stop taking your inhalers?
   Always (1) Mostly (2) Sometimes (3) Rarely (4) Never (5)

6. Do you stop taking your inhalers because of fear of side effects?
   Always (1) Mostly (2) Sometimes (3) Rarely (4) Never (5)

7. Do you stop taking your inhalers because of considering they are useless to treat your condition?
   Always (1) Mostly (2) Sometimes (3) Rarely (4) Never (5)

8. Do you take fewer inhalations than those prescribed by your doctor?
   Always (1) Mostly (2) Sometimes (3) Rarely (4) Never (5)

9. Do you stop taking your inhalers because you believe they interfere with your everyday or working life?
   Always (1) Mostly (2) Sometimes (3) Rarely (4) Never (5)

10. Do you stop taking your inhalers because you have difficulties to pay them?
    Always (1) Mostly (2) Sometimes (3) Rarely (4) Never (5)

Health care professional domain: questions, responses (scores)

11. Does the patient remember the prescribed regimen (dose and frequency)? (checking the medical record)
    No (1) Yes (2)

12. The technique of using the evaluated inhaler device by the patient is* (checking the inhalation technique)
    With critical* mistakes (1) Without critical mistakes (2)

*Critical mistakes: 1. Pressure metered dose inhalers (pMDIs): do not remove the cover, do not hold the inhaler in a vertical position, firing the device before beginning inspiration, inhalation stopped, inhalation too fast, incorrect insertion of MDI into the inhaler camera, several device firing in the same inhalation, no breath hold after inhalation, cough during inhalation. 2. Dry power inhalers (DPIs): do not open the inhaler, do not prime properly, place the device down after preparation of the dose (before inhalation), blow into the device before inhalation, inhalation not deeply and forcefully, no breath hold after inhalation.

The Morisky Scale (MMAS-8)

Is a structured self-report questionnaire composed of a total of 8 questions related to the use of medication as prescribed by a patient’s physician: 7 Yes (score, 1) and No (score, 0) questions and 1 question with 5 possible answers possible answers Likert scale type (0-4).

Morisky DE, Ang A, Krousel-Wood M, Ward HJ. Predictive validity of a medication adherence measure in an outpatient setting. J Clin Hypertens (Greenwich). 2008; 10: 348–354.

7.6 APPENDIX 6: COPD ASSESSMENT TEST (CAT)

How is your COPD? Take the COPD Assessment Test™ (CAT)

This questionnaire will help you and your healthcare professional measure the impact COPD (Chronic Obstructive Pulmonary Disease) is having on your wellbeing and daily life. Your answers, and test score, can be used by you and your healthcare professional to help improve the management of your COPD and get the greatest benefit from treatment.

For each item below, place a mark (X) in the box that best describes you currently. Be sure to only select one response for each question.

Example: I am very happy (0) (X) (2) (3) (4) (5) I am very sad

			SCORE
I never cough	0 1 2 3 4 5	I cough all the time	
I have no phlegm (mucus) in my chest at all	0 1 2 3 4 5	My chest is completely full of phlegm (mucus)	
My chest does not feel tight at all	0 1 2 3 4 5	My chest feels very tight	
When I walk up a hill or one flight of stairs I am not breathless	0 1 2 3 4 5	When I walk up a hill or one flight of stairs I am very breathless	
I am not limited doing any activities at home	0 1 2 3 4 5	I am very limited doing activities at home	
I am confident leaving my home despite my lung condition	0 1 2 3 4 5	I am not at all confident leaving my home because of my lung condition	
I sleep soundly	0 1 2 3 4 5	I don't sleep soundly because of my lung condition	
I have lots of energy	0 1 2 3 4 5	I have no energy at all	

TOTAL SCORE

COPD Assessment Test and CAT logo is a trademark of the GlaxoSmithKline group of companies.
© 2009 GlaxoSmithKline. All rights reserved.

7.7 APPENDIX 7: INTERNATIONAL PHYSICAL ACTIVITY QUESTIONNAIRE (IPAQ, August 2002)

IPAQ: SHORT LAST 7 DAYS SELF-ADMINISTERED FORMAT

We are interested in finding out about the kinds of physical activities that people do as part of their everyday lives. The questions will ask you about the time you spent being physically active in the **last 7 days**. Please answer each question even if you do not consider yourself to be an active person. Please think about the activities you do at work, as part of your house and yard work, to get from place to place, and in your spare time for recreation, exercise or sport.

Think about all the **vigorous** activities that you did in the **last 7 days**. **Vigorous** physical activities refer to activities that take hard physical effort and make you breathe much harder than normal. Think *only* about those physical activities that you did for at least 10 minutes at a time.

1. During the **last 7 days**, on how many days did you do **vigorous** physical activities like heavy lifting, digging, aerobics, or fast bicycling?

   _____ **days per week**

   ☐ No vigorous physical activities → ***Skip to question 3***

2. How much time did you usually spend doing **vigorous** physical activities on one of those days?

   _____ **hours per day**

   _____ **minutes per day**

   ☐ Don't know/Not sure

Think about all the **moderate** activities that you did in the **last 7 days**. **Moderate** activities refer to activities that take moderate physical effort and make you breathe somewhat harder than normal. Think only about those physical activities that you did for at least 10 minutes at a time.

3. During the **last 7 days**, on how many days did you do **moderate** physical activities like carrying light loads, bicycling at a regular pace, or doubles tennis? Do not include walking.

   _____ **days per week**

   ☐ No moderate physical activities → ***Skip to question 5***

4. How much time did you usually spend doing **moderate** physical activities on one of those days?

_____ **hours per day**

_____ **minutes per day**

☐ Don't know/Not sure

Think about the time you spent **walking** in the **last 7 days**. This includes at work and at home, walking to travel from place to place, and any other walking that you might do solely for recreation, sport, exercise, or leisure.

5. During the **last 7 days**, on how many days did you **walk** for at least 10 minutes at a time?

_____ **days per week**

☐ No walking → ***Skip to question 7***

6. How much time did you usually spend **walking** on one of those days?

_____ **hours per day**

_____ **minutes per day**

☐ Don't know/Not sure

The last question is about the time you spent **sitting** on weekdays during the **last 7 days**. Include time spent at work, at home, while doing course work and during leisure time. This may include time spent sitting at a desk, visiting friends, reading, or sitting or lying down to watch television.

7. During the **last 7 days**, how much time did you spend **sitting** on a **week day**?

_____ **hours per day**

_____ **minutes per day**

☐ Don't know/Not sure

This is the end of the questionnaire, thank you for participating.

8. SIGNATURES

ASTRAZENECA SIGNATURE(S)

Latin American Study of 24-hs Symptoms in Chronic Obstructive Pulmonary Disease (COPD) Patients (LASSYC Study)

An observational, multinational, cross sectional primary data collection study to describe symptoms around 24-hs and their relationship with adherence to respiratory treatment, direct costs and PRO in stable COPD patients in Latin America

This NIS Protocol of LASSYC Study has been subjected to an internal AstraZeneca review.
I agree to the terms of this Non-Interventional Study protocol

AstraZeneca representative

Filip Sumont
Medical Director AZ LatAm
filip.surmont@astrazeneca.com
+1 302 4151561

Date
(Day Month Year)

This document contains confidential information, which should not be copied, referred to, released or published without written approval from AstraZeneca. Investigators are cautioned that the information in this protocol may be subject to change and revision.

ASTRAZENECA SIGNATURE(S)

Latin American Study of 24-hs Symptoms in Chronic Obstructive Pulmonary Disease (COPD) Patients (LASSYC Study)

An observational, multinational, cross sectional primary data collection study to describe symptoms around 24-hs and their relationship with adherence to respiratory treatment, direct costs and PRO in stable COPD patients in Latin America

This NIS Protocol of LASSYC Study has been subjected to an internal AstraZeneca review.
I agree to the terms of this Non-Interventional Study protocol.

AstraZeneca representative

Valentina Di Boscio<br>*NIS Study Delivery Leader*<br>*Valentina.diboscio@astrazeneca.com*<br>*+5491167051040*	Date<br>(Day Month Year)

This document contains confidential information, which should not be copied, referred to, released or published without written approval from AstraZeneca. Investigators are cautioned that the information in this protocol may be subject to change and revision.

SIGNATURE OF INTERNATIONAL CO-ORDINATING INVESTIGATOR

Latin American Study of 24-hs Symptoms in Chronic Obstructive Pulmonary Disease (COPD) Patients (LASSYC Study)

An observational, multinational, cross sectional primary data collection study to describe symptoms around 24-hs and their relationship with adherence to respiratory treatment, direct costs and PRO in stable COPD patients in Latin America

This NIS Protocol of LASSYC Study has been subjected to an internal AstraZeneca review.
I agree to the terms of this Non-Interventional Study protocol

I agree to the terms of this Non-Interventional Study protocol. I will conduct the study according to the procedures specified herein, and according to the local regulations.

Signature:

Marc Miravitlles<br>*Chairman Scientific Committee*<br>*marcm@separ.es*<br>*+34 93 2263911*	Date<br>(Day Month Year)

This document contains confidential information, which should not be copied, referred to, released or published without written approval from AstraZeneca. Investigators are cautioned that the information in this protocol may be subject to change and revision.

SIGNATURE OF INTERNATIONAL CO-ORDINATING INVESTIGATOR

Latin American Study of 24-hs Symptoms in Chronic Obstructive Pulmonary Disease (COPD) Patients (LASSYC Study)

An observational, multinational, cross sectional primary data collection study to describe symptoms around 24-hs and their relationship with adherence to respiratory treatment, direct costs and PRO in stable COPD patients in Latin America

This NIS Protocol of LASSYC Study has been subjected to an internal AstraZeneca review.
I agree to the terms of this Non-Interventional Study protocol

I agree to the terms of this Non-Interventional Study protocol. I will conduct the study according to the procedures specified herein, and according to the local regulations.

Signature:

María Montes de Oca
Chairman Scientific Committee
montesdeoca.maria@gmail.com
+58 414 3314148

Date
(Day Month Year)

This document contains confidential information, which should not be copied, referred to, released or published without written approval from AstraZeneca. Investigators are cautioned that the information in this protocol may be subject to change and revision.

SIGNATURE OF INTERNATIONAL CO-ORDINATING INVESTIGATOR

Latin American Study of 24-hs Symptoms in Chronic Obstructive Pulmonary Disease (COPD) Patients (LASSYC Study)

An observational, multinational, cross sectional primary data collection study to describe symptoms around 24-hs and their relationship with adherence to respiratory treatment, direct costs and PRO in stable COPD patients in Latin America

This NIS Protocol of LASSYC Study has been subjected to an internal AstraZeneca review.
I agree to the terms of this Non-Interventional Study protocol

I agree to the terms of this Non-Interventional Study protocol. I will conduct the study according to the procedures specified herein, and according to the local regulations.

Signature:

Ana Baptista Menezes
Epidemiologist Statistician provider
anamene.epi@gmail.com
+ 55 53 32841300

Date
(Day Month Year)

This document contains confidential information, which should not be copied, referred to, released or published without written approval from AstraZeneca. Investigators are cautioned that the information in this protocol may be subject to change and revision.

SIGNATURE OF PRINCIPAL INVESTIGATOR

Latin American Study of 24-hs Symptoms in Chronic Obstructive Pulmonary Disease (COPD) Patients (LASSYC Study)

An observational, multinational, cross sectional primary data collection study to describe symptoms around 24-hs and their relationship with adherence to respiratory treatment, direct costs and PRO in stable COPD patients in Latin America

This NIS Protocol of LASSYC Study has been subjected to an internal AstraZeneca review.
I agree to the terms of this Non-Interventional Study protocol

I agree to the terms of this Non-Interventional Study protocol. I will conduct the study according to the procedures specified herein, and according to the local regulations..

Site No.: <<This may be hand-written onto the page at the time the signature is collected>>

Signature:

<<Name, title, email address and telephone number>>

Date
(Day Month Year)

This document contains confidential information, which should not be copied, referred to, released or published without written approval from AstraZeneca. Investigators are cautioned that the information in this protocol may be subject to change and revision.

9. LIST OF REFERENCES

1 Global Strategy for the Diagnosis, Management and Prevention of COPD, Global Initiative for Chronic Obstructive Lung Disease (GOLD) 2015. http://www.goldcopd.org. Vestbo J, et al. Am J Respir Crit Care Med. 2013 Feb 15;187(4):347-65.

2 Lopez AD, Shibuya K, Rao C, Mathers CD, Hansell AL, Held LS, Schmid V, Buist S. Chronic obstructive pulmonary disease: current burden and future projections. Eur Respir J 2006;27:397-412.

3 Buist AS, McBurnie MA, Vollmer WM, Gillespie S, Burney P, Mannino DM, Menezes AM, Sullivan SD, Lee TA, Weiss KB, Jensen RL, Marks GB, Gulsvik A, Nizankowska-Mogilnicka E; BOLD Collaborative Research Group. International variation in the prevalence of COPD (the BOLD Study): a population-based prevalence study. Lancet 2007;370:741-50.

4 Menezes AMB, Perez-Padilla R, Jardim JRB, Muiño A, Lopez MV, Valdivia G, et al. Chronic obstructive pulmonary disease in five Latin American cities (the PLATINO study): a prevalence study. Lancet. 2005;366:1875–81.

5 Caballero A, Torres-Duque CA, Jaramillo C, Bolívar F, Sanabria F, Osorio P, et al. Prevalence of COPD in five Colombian cities situated at low, medium, and high altitude (PREPOCOL study). Chest. 2008;133(2):343–9.

6 Tálamo C, de Oca MM, Halbert R, Perez-Padilla R, Jardim JRB, Muiño A, et al. Diagnostic labeling of COPD in five Latin American cities. Chest. 2007;131(1):60–7

7 Perez-Padilla R, Wehrmeister FC, Celli BR, Lopez-Varela MV, Montes de Oca M, Muiño A, et al. Reliability of FEV1/FEV6 to diagnose airflow obstruction compared with FEV1/FVC: the PLATINO longitudinal study. PloS One. 2013;8(8):e67960

8 Miravitlles M, Anzueto A, Legnani D, Forstmeier L, Fargel M. Patient's perception of exacerbations of COPD – the PERCEIVE study. Respir Med 2007;101:453-60.

9 Carrasco Garrido P, de Miguel Díez J, Rejas Gutiérrez J, Centeno AM, Gobartt Vázquez E, Gil de Miguel A, García Carballo M, Jiménez García R.. Negative impact of chronic obstructive pulmonary disease on the health-related quality of life of patients. Results of the EPIDEPOC study. Health Qual Life Outcomes 2006;4:31.

10 Partridge MR, Karlsson N, Small IR. Patient insight into the impact of chronic obstructive pulmonary disease in the morning: an internet survey. Curr Med Res Opin 2009;25:2043-8.

11 Kessler R, Partridge MR, Miravitlles M, Cazzola M, Vogelmeier C, Leynaud D, Ostinelli J. Symptom variability in patients with severe COPD: a pan-European cross-sectional study. Eur Respir J 2011;37:264-72.

12 Ferrer M, Alonso J, Morera J, Marrades RM, Khalaf A, Aguar MC, Plaza V, Prieto L, Antó JM. Chronic obstructive pulmonary disease stage and health-related quality of life. The Quality of Life of Chronic Obstructive Pulmonary Disease Study Group. Ann Intern Med 1997;127:1072-9.

13 Hajiro T, Nishimura K, Tsukino M, Ikeda A, Koyama H, Izumi T. Comparison of discriminative properties among disease-specific questionnaires for measuring health-related quality of life in patients with chronic obstructive pulmonary disease. Am J Respir Crit Care Med 1998;157:785-90.

14 Plaza V, Fernandez-Rodriguez C, Melero C, Cosıo B et al. Validation of the ‘Test of the Adherence to Inhalers’(TAI) for Asthma and COPD Patients. Journal of Aerosol medicine and Pulmonary Drud Delivery 2015; 28:2-11

15 Global Strategy for Asthma management and Prevention – GINA- 2015 Report. www.ginasthma.org

16 García-Aymerich J, Serra I, Gómez FP, Farrero E, Balcells E, Rodríguez DA, de Batlle J, Gimeno E, Donaire-Gonzalez D, Orozco-Levi M, Sauleda J, Gea J, Rodriguez-Roisin R, Roca J, Agustí AG, Antó JM; Phenotype and Course of COPD Study Group. Physical activity and clinical and functional status in COPD. Chest 2009;136:62-70.

17 Jones PW. Activity limitation and quality of life in COPD. COPD. 2007;4:273-8.

18 Benzo RP, Chang CC, Farrell MH, Kaplan R, Ries A, Martinez FJ, Wise R, Make B, Sciurba F; NETT Research Group. Physical activity, health stauts and risk of hospitalization in patients with severe chronic obstructive pulmonary disease. Respiration 2010;80:10–8.

19 García-Aymerich J, Lange P, Benet M, Schnohr P, Antó JM. Regular physical activity reduces hospital admission and mortality in chronic obstructive pulmonary disease: a population-based cohort study. Thorax 2006;61:772-8.

20 Glaab T, Vogelmeier C, Buhl R. Outcome measures in chronic obstructive pulmonary disease (COPD): strengths and limitations. Respir Res 2010;11:79.

21 Klink M, Quan SF. Prevalence of reported sleep disturbances in a general adult population and their relationship to obstructive airways diseases. Chest 1987;91:540-6.

22 Divo M, Cote C, de Torres JP, Casanova C et al for the BODE Collaborative Group. Comorbidities and risk of mortality in patients with chronic obstructive pulmonary disease. Am J Respir Crit Care Med. 2012; Vol 186 (2): 155–161.

23 Miravitlles M, Worth H, Soler Cataluña JJ, Price D et al. Observational study to characterize 24-hour COPD symptoms and their relationship with patient-reported outcomes: results from the ASSESS Study. Respiratory research 2014; 15: 122-134.

24 National heart Lung and Blood Institute. Morbidity and mortality chart book on cardiovascular lung and blood diseases. Bethesda Maryland: US Department of Health and Human Services. Public Health Service, National Institute of health. www.nhlbi.nih.gov/resources/docs/cht-book.htm; 2009.

25 Soler-Cataluña JJ, Martinez-Garcia MA, Roman P, Salcedo E, Navarro M, Ochando R. Severe acute exacerbations and mortality in patients with chronic obstructive pulmonary disease. Thorax 2005;60:925e31.

26 Regueira CR, Hamel MB, Davis RB, Desbiens N et al. A comparison of generalist and pulmonologist care for patients hospitalized with severe chronic obstructive pulmonary disease: resource intensity, hospital costs and survival. Am J Med 1998; 105:366-72

27 International Society for Pharmacoepidemiology. Guidelines for Good Pharmacoepidemiology Practices (GPP). Revision 2: April 2007 http://pharmacoepi.org/resources/guidelines_08027.cfm.

28 International Society for Pharmacoeconomics and Outcomes Research. Prospective Observational Clinical Studies Good Research Practices Task Force. http://www.ispor.org/TaskForces/ObservationalClinicalStudyGRP.asp.

29 Bestall JC, Paul EA, Garrod R, Garnham R, Jones PW, Wedzicha JA. Usefulness of the Medical Research Council (MRC) dyspnoea scale as a measure of disability in patients with chronic obstructive pulmonary disease. Thorax 1999;54:581-6.

30 Bourbeau J, Ford G, Zackon H, Pinsky N, Lee J, Ruberto G. Impact on patients' health status following early identification of a COPD exacerbation. Eur Respir J 2007;30:907-13.

31 de Torres JP, Pinto-Plata V, Ingenito E, Bagley P, Gray A, Berger R, Celli B. Power of outcome measurements to detect clinically significant changes in pulmonary rehabilitation of patients with COPD. Chest 2002;121:1092-8.

32 Soler Cataluña JJ, Martinez-Garcia MA, Sanchez Sanchez L, Perpiña Tordera M and Roman Sanchez P. Severe exacerbations and BODE index: two independent risk factors for death in male COPD patients. Respiratory Medicine. 2009; 103:692-699.

33 Celli BR, Cote CG, Marín JM, et al. The body-mass index, airflow obstruction, dyspnea, and exercise capacity index in chronic obstructive pulmonary disease. N Engl J Med 2004;350:1005-12.

34 Jones PW, Harding G, Berry P, Wiklund I, Chen WH, Kline Leidy N. Development and first validation of the COPD Assessment Test. Eur Respir J 2009;34:648-54.

35 Ainsworth BE, Bassett DR Jr, Strath SJ, Swartz AM, O'Brien WL, Thompson RW, Jones DA, Macera CA, Kimsey CD. Comparison of three methods for measuring the time spent in physical activity. Med Sci Sports Exerc 2000;32:S457-64.

36 Morisky DE, Green LW, Levine DM. Concurrent and predictive validity of a self-reported measure of medication adherence. Med Care.1986;24 (1):67-74.

37 Ivanova J, Birnbaum H, Hsieh M, Yu M et al. Adherence to inhaled corticosteroid use and local adverse events in persistent asthma. American Journal Managed Care 2008; 14(12) 801-809.

38 Brooks MC, Richards JM, Kohler CL, et al. Assessing adherence to asthma medication and inhaler regimens: a psychometric analysis of adult self-reported scales. Med Care. 1994;32(3):298-307.
